# Worry and rumination elicit similar neural representations: neuroimaging evidence for repetitive negative thinking

**DOI:** 10.3758/s13415-024-01239-z

**Published:** 2024-11-19

**Authors:** Nikki A. Puccetti, Caitlin A. Stamatis, Kiara R. Timpano, Aaron S. Heller

**Affiliations:** 1 Department of Psychiatry, The Ohio State University Wexner Medical Center, 1670 Upham Dr, Columbus, OH 43210 USA; 2 Department of Psychology, University of Miami, PO Box 248185, Coral Gables, FL 33124 USA; 3 Department of Preventative Medicine, Northwestern Feinberg School of Medicine, Chicago, IL USA; 4 Bruin Health Inc., New York, NY USA

**Keywords:** fMRI, Representational similarity analysis, Transdiagnostic vulnerability, Peseverative cognition

## Abstract

Repetitive negative thinking (RNT) captures shared cognitive and emotional features of content-specific cognition, including future-focused worry and past-focused rumination. The degree to which these distinct but related processes recruit overlapping neural structures is undetermined, because most neuroscientific studies only examine worry or rumination in isolation. To address this, we developed a paradigm to elicit idiographic worries and ruminations during an fMRI scan in 39 young adults with a range of trait RNT scores. We measured concurrent emotion ratings and heart rate as a physiological metric of arousal. Multivariate representational similarity analysis revealed that regions distributed across default mode, salience, and frontoparietal control networks encode worry and rumination similarly. Moreover, heart rate did not differ between worry and rumination. Capturing the shared neural features between worry and rumination throughout networks supporting self-referential processing, memory, salience detection, and cognitive control provides novel empirical evidence to bolster cognitive and clinical models of RNT.

**Supplementary information:**

The online version contains supplementary material available at 10.3758/s13415-024-01239-z.

## Introduction

Repetitive negative thinking (RNT) refers to perseverative thoughts about negative aspects of the self, environment, and experiences (Ehring & Watkins, 2008). Conceptually, RNT is a latent construct reflecting a shared cognitive process among content-specific types of thinking, such as worry and rumination (Arditte et al., 2016). Worry—uncontrollable and exaggerated negative thoughts about *future* catastrophes (Borkovec & Inz, 1990)—is traditionally associated with anxiety disorders, whereas rumination—dwelling on *past* problems and failures (Nolen-Hoeksema et al., 2008)—is traditionally associated with depressive disorders. The separation of worry and rumination in the clinical literature extends to the neuroscientific literature with little research testing whether the neural representation of worry and rumination processes overlap. Testing whether worry and rumination overlap neurobiologically would help understand the degree to which RNT is indeed a shared cognitive process. Moreover, identifying specific brain circuits underlying RNT is important to improve etiological and treatment models of psychological distress across diagnostic categories.

The disparate lines of research on worry and rumination implicate regions from three functional brain networks: the default mode, lateral frontoparietal, and salience networks (Menon, 2011; Koster et al., 2011; Lydon Staley et al., 2019). The functions of these networks map onto common features among worry and rumination, suggesting that they likely recruit similar neural structures. For example, within the default mode network (or medial frontoparietal network; Uddin et al., 2019), involvement of the posterior cingulate cortex (PCC) and the medial prefrontal cortex (PFC) in worry and rumination likely reflects increased self-relevant and autobiographical processing that is characteristic of RNT (Andrews-Hanna et al., 2014; Buckner & Carroll, 2007; Cooney et al., 2010; Nejad et al., 2013; Paulesu et al., 2010; Servaas et al., 2014; Zhou et al., 2020). Involvement of the lateral frontoparietal network, specifically the dorsolateral PFC, in worry and rumination suggests difficulty guiding the inhibition of, and redirection from, negative thoughts during RNT (Cooney et al., 2010; Hamilton et al., 2011; Kühn et al., 2012; Miller & Cohen, 2001; Niendam et al., 2012; Servaas et al., 2014). Finally, involvement of salience network (or midcingulo-insular network; Uddin et al., 2019) regions, including the anterior cingulate cortex (ACC) and insula, in worry and rumination may indicate a heightened salience of negative endogenous stimuli (i.e., thoughts and memories) and feeling “stuck” in RNT (Andreescu et al., 2011; Burrows et al., 2017; Kalisch & Gerlicher, 2014; Kühn et al., 2012; Nejad et al., 2013; Paulesu et al., 2010; Piguet et al., 2014; Servaas et al., 2014).

Despite the evidence of conceptual similarities between worry and rumination, few studies have investigated the extent and spatial pattern of their neural similarity in the same sample. One study (Steinfurth et al., 2017) found that state worry and rumination during fMRI were similarly different from the neutral prompts provided across regions of the salience, default mode, and lateral frontoparietal networks, while also retaining some unique activation. However, this study was limited by relatively few trials and a lack of concurrent biological and subjective markers of emotional experience. Moreover, the studies reviewed are limited by a reliance on traditional univariate analyses that aggregate across voxels and stimuli to quantify the magnitude of signal across a brain region. This approach does not capture the nuanced information about stimulus features that is encoded in patterns of activity across voxels, which can distinguish stimuli that otherwise evoke identical mean activation (Kriegeskorte & Bandettini, 2007). Thus, to determine the degree to which the brain represents worry and rumination’s shared features, it is crucial to examine the multivoxel patterns of activation elicited by many personal worries and ruminations in the same sample.

The current study investigated the overlap in neural representations by using multivoxel analysis of idiographic worry and rumination inductions. A sample of young adults with a range of trait RNT wrote personal worry and ruminations outside of the fMRI environment. These statements were presented during a later fMRI paradigm, where participants mulled them over along with neutral statements. We conceptualized the overlap in the neural representations of worries and ruminations as the shared process of RNT and quantified this overlap using multivariate representational similarity analysis (RSA). Unlike univariate modeling of individual voxels, RSA compares the patterns of activation across a group of voxels, which carry rich information about unique stimuli (Averbeck et al., 2006; Kriegeskorte & Kievit, 2013). Using RSA, we calculated the similarity in neural patterns during worry and rumination to determine the representational distance between them. Higher similarity, or shorter representational distance, supports the notion of a common process between worry and rumination. We used a searchlight procedure to identify where participants’ worries and ruminations are represented most similarly to one another, yet differently from neutral states. In line with previous work (Koster et al., 2011; Menon, 2011; Burrows et al., 2017; Lydon Staley et al., 2019), we hypothesized that worry and rumination would show similar activity patterns in the default, frontoparietal, and salience networks.

We then tested the specificity of the primary RSA relative to general negative affect. Specifically, we tested whether neural structures distinguishing worry and rumination from neutral prompts resembled those distinguishing standardized, visual, and nonautobiographical negative and neutral stimuli. To this end, a subset of participants viewed negative and neutral International Affective Picture System (IAPS; Lang et al., 2008) images during fMRI. We conducted a similar searchlight RSA distinguishing negative and neutral images, hypothesizing that the worry and rumination effects would be at least partly unique from the IAPS effects.

## Methods

### Participant characteristics

**Fig. 1:**
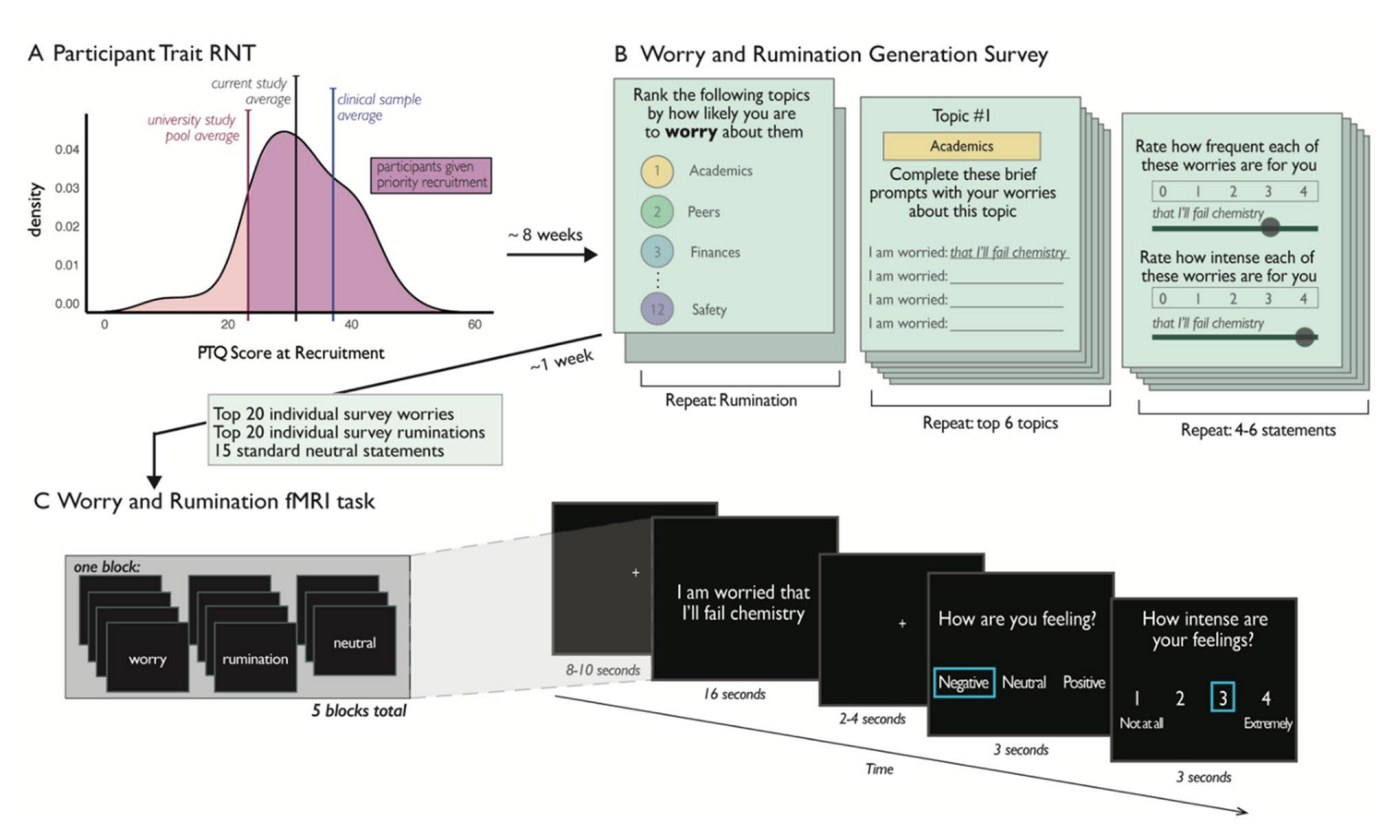
Worry and rumination paradigm. **A)** We oversampled for participants with trait RNT higher than their peers (study pool M = 23) at the time of recruitment. Thus, our sample mean (31) fell between our average student and clinical samples that tend to report even higher RNT (M = 37). **B)** Participants viewed a list of 13 topics and ranked them by how much they worry about that topic. For each of their top 6 topics, they provide 4 to 6 specific worries and rate their frequency and intensity. They repeat this for rumination. **C)** The top 20 most intense worries and 20 ruminations are combined with 15 neutral statements and used as the stimuli in the fMRI task approximately 1 week later. Participants are asked to re-engage with their statements and mull them over for 16 s and then rate the valence and intensity of their emotion experience (3 s each) while engaging with the thoughts

A total of 152 undergraduate participants from the University of Miami were recruited between 2017–2019 from an online study pool at the beginning of the academic semester. In general college populations, trait RNT levels can be positively skewed with fewer individuals on the high end of the distribution (Devynck et al., 2017). Because the current study required participants to generate then eventually recall and perseverate on their day-to-day worries and ruminations, we wanted to ensure that we did not recruit too many individuals with minimal RNT. For this reason, we prioritized recruitment of students reporting higher-than-average RNT during a pre-semester assessment battery. We computed the mean score on the Perseverative Thinking Questionnaire (PTQ; Ehring et al., 2011) across a large pool of approximately 600 psychology students (mean (*M*) = 23, standard deviation (SD) = 12.41). From this pool, students with a score above the mean were sent individual messages inviting them to participate and given priority scheduling. Other students were eligible and able to sign up for the current study that appeared in their online portal, but they were not personally invited to the study. This oversampling resulted in our sample’s mean PTQ score falling between the larger student sample and reported clinical samples of depressed and anxious patients (Fig. 1).

All participants completed a battery of questionnaires measuring worry, rumination, and other mental health symptoms at the beginning of the semester (time 1), and 120 of those 152 people repeated these questionnaires at the end of the semester (time 2). At time 2, participants also completed the worry and rumination generation survey. The current study focused on the 39 participants that not only completed time 2, but who also indicated their interest and met eligibility criteria (i.e., no metal in the body) for an additional study visit that included fMRI. This group of participants did not differ from those who were not scanned other than they happened to report higher trait RNT at time 1 (Supplemental Table 1 shows the full group comparisons). All 39 participants completed their scan approximately 1 week after the time 2 visit. To test the specificity of the primary RSA relative to general negative affect, a subset of 23 participants also completed an IAPS viewing task; due to time constraints during the scan session (of the 39 scanned, 10 participants did not complete the IAPS task and 6 only completed 1 of 2 blocks). Participants were compensated with a mix of research familiarization credits and cash. Demographic information and descriptive statistics for the self-report measures are displayed in Table 1.

**Table 1:** Sample demographics and descriptive statistics for trait RNT at the end of the semester

	**Mean**	**SD**
Age	18.8	0.84
	**N (of 39)**	**%**
Female	22	57
Male	17	43
*Ethnicity:*		
Hispanic	11	28
*Race:*		
African American/Black	4	10
Asian/Asian-American	8	21
Caucasian/White	21	54
Other	2	5
No response	4	10
	**Mean**	**SD**
RNT (PTQ)	26.3	9.8

### Self-report measures

*The Perseverative Thinking Questionnaire (PTQ).* The PTQ is a 15-item questionnaire that measures the tendency to engage in repetitive negative thinking, particularly when encountering problems (Ehring et al., 2011). The items are rated on a scale from “0” (never) to “4” (almost always). Item ratings were summed to create each participant’s score.

*Worry and Rumination Generation Survey.* First, participants learned the definitions of worry and rumination (worry emphasized as future-focused and rumination as past-focused; see Supplement for exact definitions and prompts) and then demonstrated their understanding by correctly classifying fictional scenarios that describe characters either worrying or ruminating. See the supplemental methods for the text provided to participants in this survey and detailed experimental protocol. Participants then ranked a list of 13 topics by how much they worry about them (topics included school, friends, job, health, finances, romance, world events, parents, family, safety, roommates, achievement, and other). For each of their top 6 topics, participants wrote 4 to 6 idiographic worry statements about that topic and rated their frequency and intensity from 0, “Not at all,” to 4, “Extremely.” Participants repeated the topic ranking, statement generation, and ratings for rumination (Fig. 1).

### fMRI procedure and acquisition

*Worry and Rumination Generation fMRI Task Design*. During this paradigm, participants reflected on 20 worry statements, 20 rumination statements, and 15 neutral statements (Fig. 1). The idiographic worry and rumination statements used were the 20 survey statements that participant generated and rated as most intense 1 week prior. The 15 neutral statements were generated by experimenters to convey brief, relatively non-emotive, yet self-referential scenarios (i.e., “I sometimes think about different routes to take to class”). This was done because we believed it would be difficult for participants to generate their own neutral, atemporal, self-relevant statements as an appropriate control. Participants knew which type of statement was presented each trial, because the worry statements began with “I am worried…”, rumination statements began with “I often think back on…”, and neutral statements began with “I sometimes think about.” For a full list of neutral statements and example worry and rumination statements, see Supplemental Table 6.

Before completing the fMRI task, participants viewed their top 20 worry and 20 rumination statements to confirm that participants 1) remembered each statement, 2) felt it was still relevant, and 3) perceived the statements as generally negative worries or ruminations. If any statements violated these characteristics, the participant wrote new, appropriate statements before the scan (27 statements across 4 unique participants were changed). Participants completed one practice block with lower intensity survey statements that were not used for the main task. The supplement describes this protocol in depth.

Participants were informed that they would see their idiographic worry and ruminations, as well as other thoughts people sometimes have, one at a time. Participants were instructed to think about each statement “as intensely as you can, for as long as it is presented” and to “immerse yourself in the statement and re-experience it as vividly and emotionally as you can.” Participants completed 5 functional runs with 4 worry, 4 rumination, and 3 neutral statement trials in random order. Each run began with a 30-s white fixation cross on a black background. Each trial began with either an 8-s or 10-s fixation cross before displaying the statement for 16 s in white font on a black screen (see Fig. 1 for task schematic). After, a fixation cross appeared for either 2 s or 4 s, followed by two rating screens lasting 3 s each: first, participants used a button box to indicate whether they were feeling “Negative,” “Neutral,” or “Positive,” when reengaging with the thought; then, participants rated how intense their feelings were when reengaging with the thought on a scale from 1 to 4.

*fMRI IAPS Task Design.* Participants completed the IAPS task after the Worry and Rumination Generation Task. Participants were instructed to view 20 negative and 20 neutral IAPs images in a pseudo random order across two blocks. We specifically chose the 20 highest arousal negative images and the 20 most neutral images based on the standardized ratings. Each run began with a 24-s white fixation cross on a black background. Each trial began with a fixation cross for 8 s or 10 s, followed by an image displayed for 4 s, followed by a fixation cross for 2 s or 4 s.

*Scan Acquisition.* MR data were acquired with a GE Discovery MR750 3 T scanner. A standard clinical whole-head quadrature head coil was used. Five functional runs for the worry and rumination generation task were collected. For participants who completed the IAPS task, an additional two functional runs were collected. Functional images were obtained using a T2*-weighted, echo-planar images (EPI); 38 sagittal slices, 3.4-mm thickness with 3.4-mm gap; 3.43 × 3.43 mm in-plane (64 × 64 matrix); FOV = 220; repetition time (TR)/echo time (TE)/Flip = 2000 ms/30 ms/75°; 208 whole-brain volumes per run. Additionally, a high-resolution T1-weighted anatomical image was obtained: T1-weighted inversion recovery fast gradient echo with 188 axial slices, 1-mm thickness; 256 × 256 in-plane resolution.

### fMRI data preprocessing

Data were preprocessed using fMRIPrep 1.4.1 (Esteban, Markiewicz et al. (2018); Esteban, Blair et al. (2018), a standardized preprocessing pipeline for fMRI data that is based on Nipype 1.2.0 (Gorgolewski et al., 2011). fMRIPrep encourages users to report a semi-standardized description of the analyses generated directly from the program, which we include in the supplement.

### Concurrent Psychophysiology acquisition during fMRI

For each fMRI task run, we acquired psychophysiological data, including heart rate. These data were collected using the Biopac MP160 data acquisition system. Heart rate was captured using the PPG100C-MRI Photo Plethysmogram amplifier which recorded from the pad of the participant’s ring finger on their left hand with a sampling rate of 1000 Hz. These data were converted from the BioPac AcqKnowledge program (version 4.3.1) for analysis in Mindware 3.2. Heart rate time series were segmented into 34-s epochs that aligned with each fMRI trial. Peaks were automatically labelled, manually checked by research assistants, and double-checked by the first author.

### Behavioral and physiological data analysis

To validate that participant’s wrote distinguishable worry and rumination statements, we tested whether a large language model could successfully classify participant statements. Full methods and results of this process are presented in the supplement. Briefly, we provided the Mistral-7B-Instruct-v0.2 model (https://mistral.ai/) the exact instructions given to participants as well as the participants’ statements (*N* = 2078). The worry and rumination specific stems, such as “I am worried…,” were removed. Our primary outcome of interest was model precision, or the percent of statements classified as worry, for example, by the model that were indeed truly worry statements from participants.

Next, we conducted sanity checks and checked the convergent validity of the worry and rumination generation survey. We used a hierarchical regression model to confirm whether participants rated the intensity and frequency of their survey statements more highly if those statements were on topics that were ranked as more personally relevant and concerning. This model also included trait RNT as a predictor to determine whether an established measure of RNT would predict participants’ frequency and intensity ratings on the worry and rumination survey. We regressed the survey ratings on a scale of 0 to 4 made for each statement on the type of rating (intensity or frequency), topic rank (1 to 6), type of RNT (worry or rumination), and trait RNT as predictors with a random intercept for participant.

To assess agreement between intensity ratings made on the survey before the scan and those made during the fMRI task, we tested a hierarchical model that regressed statement intensity ratings from the fMRI task on the corresponding intensity rating from the survey for that same statement. This hierarchical model included both within- and between-subjects effects of survey intensity rating and a random intercept and slope for subject. Specifically, the within-subject predictor was calculated by subtracting each intensity rating from that person’s own mean and the between person predictor was that person’s mean (Curran & Bauer, 2011). We also conducted regressions to confirm that the worry and rumination statements during the fMRI scan were rated more negative and intense than the neutral statements.

Finally, we examined whether worry and rumination show similar or unique physiological profiles by assessing the relationship between heart rate and intensity rating during the fMRI. Hierarchical regression models tested whether task condition (i.e., worry, rumination, or neutral) predicted one’s heart rate on that trial. In addition to testing the effect of condition, we also tested the effect of trial-by-trial emotional intensity ratings on heart rate. The same models using heart rate variability are included in the Supplement.

### fMRI data analysis

Our primary aim was to identify the neural substrates of RNT by testing the degree of neural overlap when people were engaging in rumination or worry. We first conducted a standard univariate GLM of each worry, rumination, and neutral trial to generate the input to a multivariate searchlight RSA. Preprocessed data were input to AFNI’s 3dDeconvolve function (Cox, 1996) with each trial modelled separately alongside 6 head motion parameters. Each trial’s whole brain beta map was grouped by trial type (worry, rumination, neutral) and passed to the RSA.

Given the ubiquity of traditional univariate GLMs, we also conducted two univariate analyses independent of the RSA, which are presented in the supplement. First, we derived a worry + rumination versus neutral condition contrast. Additionally, to determine what regions’ mean activity covaried with a shared aspect of RNT, emotion intensity, we conducted an amplitude modulation analysis of each trial by the participants’ emotional intensity rating. Although these analyses were not central to our hypothesis, they complement our main analyses and aid with integration into the extant literature.

**Fig. 2:**
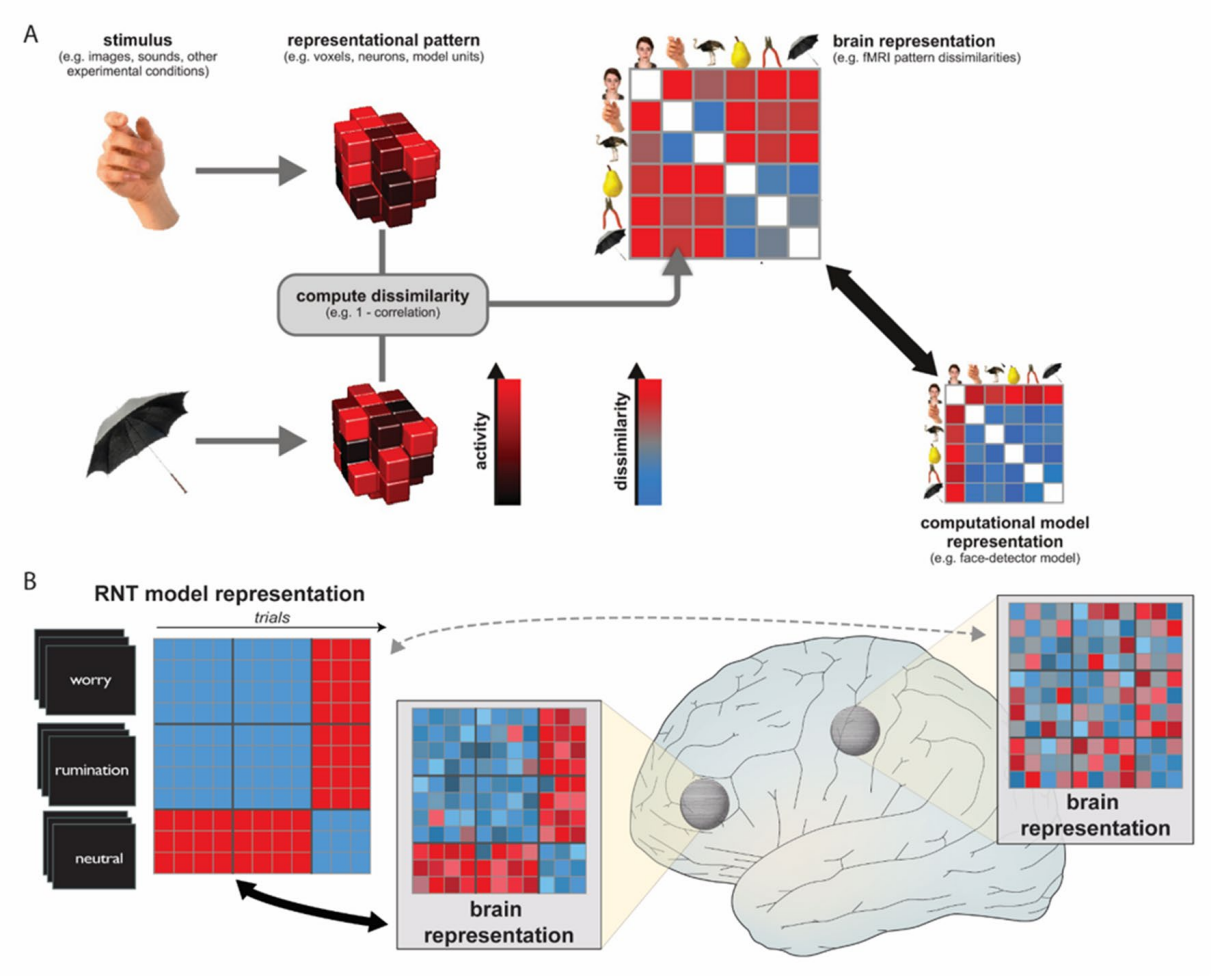
Overview of RSA for brain data and searchlight procedure for the fMRI task. **A)** From Kriegeskorte & Kievit, 2013. Shows the fundamental steps for RSA, which quantifies how related different stimulus representations are in the brain and allows the brain dissimilarity matrix to be compared with a computational or theoretical model. This comparison used in **B)** shows the searchlight that moves a sphere throughout the brain to identify clusters whose trial-by-trial dissimilarity matrix matches the model matrix (high similarity within and between worry and rumination trials but dissimilarity with neutral trials). Some clusters may significantly match the model, shown with the thick black arrow, whereas others do not, shown with the dashed gray arrow. The example models here are not showing real data nor the exact number of trials (rows and columns)

*Primary representational similarity analysis.* In this study, representational distance between each pair of trials was calculated by taking 1 minus the correlation between voxel-wise activity maps. This distance, or dissimilarity value, for all possible pairs of worry, rumination, and neutral trials was entered into a representational dissimilarity matrix (RDM; Fig. 2a) (Edelman et al., 1998; Haxby et al., 2001; Kreigeskorte, 2008). To conduct the searchlight RSA, we specified a *model* RDM reflecting our hypothesis that rumination and worry share common neural mechanisms and thus would be represented similarly (a dissimilarity or distance of 0), and moreover would be dissimilar from the neutral condition (Fig. 2b). We used a 9-mm–radius spherical searchlight across the brain to identify clusters of voxels who’s observed RDM significantly matched the model RDM. The significance of the correlation between the model and observed RDMs was determined by one-sided signed-rank test and the resulting *p*-value attributed to the central voxel of the sphere (Nili et al., 2014; Wilcoxon, 1945). This was repeated until a whole-brain map of *p*-values was obtained and corrected for multiple comparisons by thresholding at a false discovery rate of *a* = 0.05.

To test whether results from the above analysis could be attributable to confounding effects of the neutral statements (i.e., novelty), we performed an additional searchlight analysis that only included worry and rumination trials. In contrast to the primary analysis, this model RDM was defined as the maximal *dissimilarity* in neural patterns between worry and rumination trials. This direct comparison between worry and rumination, ignoring neutral statements, allows us to determine whether significant *differences* between them may have been obscured by possible confounding factors of the neutral statements.

*Specificity analysis using standardized affective images.* To test whether any of the RSA effects could be attributable to general negative affect, we performed an additional analysis where we tested whether neural structures distinguishing worry and rumination from neutral prompts resembled those distinguishing standardized, visual, nonautobiographical negative stimuli (IAPS images). By completing a RSA searchlight on negative and neutral stimuli with different features than the RNT stimuli, we tested how results may be driven by a valence effect. This analysis was constrained to the 23 participants who completed both runs of the IAPS paradigm to ensure each subject’s RDM was the same size as one another and the model RDM. Identical trial-wise GLM and RSA parameters were used, but with a new model RDM that coded negative and neutral image trials as maximally dissimilar. Resulting clusters then reflected voxels that distinguished negative from neutral images. The statistical map was FDR corrected to determine the statistical significance. Visual inspection of IAPS map overlaid on the worry and rumination RSA map was used to determine the similarity between the two.

## Results

Table 1 shows the sample demographics as well as the mean and standard deviation (SD) of trait RNT at the end of the semester. Figure 1 shows the distribution of RNT at the time of recruitment to illustrate our oversampling of people with high-than-average trait RNT. Additional descriptive statistics on sample characteristics, including trait worry, trait rumination, and depression and anxiety symptoms, are provided in Supplemental Table 1.

### The worry and rumination generation survey elicits meaningful, personally relevant worries and ruminations

We first validated our worry and rumination survey by asking a large language model to distinguished between the worry and rumination statements generated. We found that the model achieved 93% precision when instructed to classify participants’ statements as worry or rumination, indicating it was very uncommon for a worry to be classified as a rumination or vise-a-versa. The model provided the category of each statement as well as a rationale for how the statement aligned with the conceptual definitions of worry and rumination. For example, the model classified, “*If we got married our religious differences would cause marital problems*,” as a worry statement, describing its reasoning as, “*First, the speaker in the statement is considering a hypothetical situation where they get married. This is a future statement. Additionally, the potential for religious differences to cause marital problems could evoke negative thoughts and emotions for the speaker. Therefore, this statement is future-focused on a potential negative outcome, meeting the definition of worry*.” Additional examples of correct and incorrect classifications and the accompanying rationales are provided in Supplemental Table 7. This differentiation, in combination with explicit task instructions and worry- or rumination-specific statement stems (i.e., “I am worried…”), increases our confidence that participants engaged with the appropriate thought type (worry or rumination) when completing the fMRI task.

**Fig. 3:**
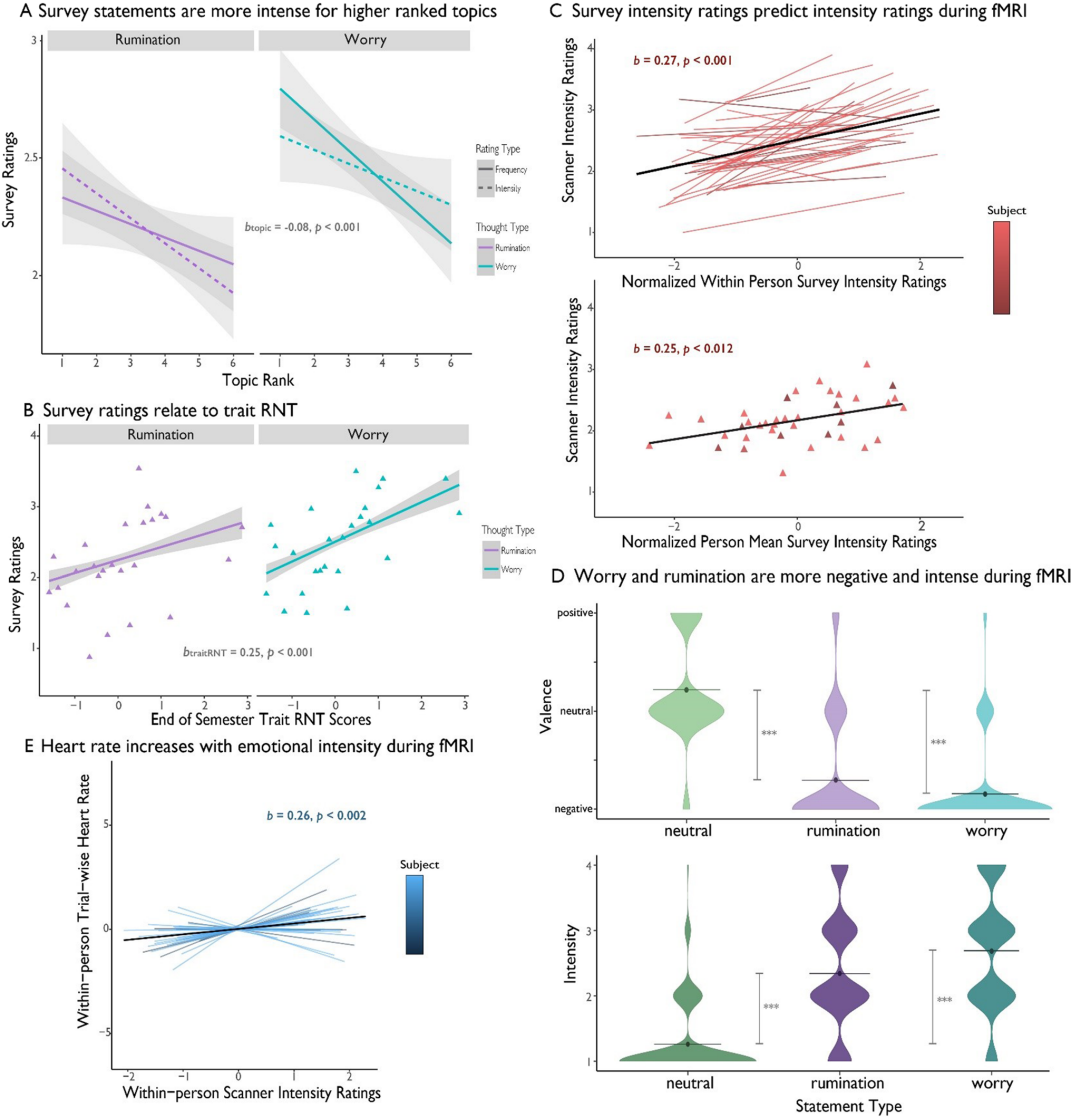
Behavioral and physiological evidence that the worry and rumination paradigm elicited negative emotional experiences. **A)** From the hierarchical model, frequency and intensity ratings from the worry and rumination generation survey were rated more intense and frequent for more highly ranked topics. Worry was also shown to be rated more highly than rumination on average. **B**) From the same model as A, participant’s frequency and intensity ratings for worry and rumination from the survey are significantly associated with their trait RNT scores from the end of the semester. **C)** Statement intensity ratings made on the worry and rumination generation survey are significantly related to intensity ratings to the same statement during the fMRI scan. The top plot shows a positive within-person effect, such that statements rated more intense than one’s average were also rated higher in the scanner; the bottom plot shows a positive between-person effect, such that participants who rated their statements higher on average on the worry and rumination survey also rated statements higher on average in the scanner. **D)** During the fMRI scan, worry and rumination were rated more negative than neutral statements and more emotionally intense than the neutral statements. **E)** During the fMRI scan, participants’ heart rate was higher on trials that were rated as more emotionally intense

We further validated the survey examining how participants’ ratings of their worry and rumination statements varied by rating type (frequency or intensity), statement type (worry or rumination), topic rank (i.e., how personally relevant; Supplemental Fig. 1 visualizes these ratings), as well as converged with trait scores of RNT. A main effect of topic rank (*b* =  − 0.08, *SE* = 0.01, *t*(803.23) =  − 6.95, *p* < 0.001) showed that participants rated statements written for the most personally relevant topics (i.e., friends) as more intense and frequent across worry and rumination (Fig. 3A). Additionally, a main effect of statement type showed that worry statements were rated more frequent and intense than rumination statements (*b* = 0.30, *SE* = 0.04, *t*(804.00.93) = 7.27, *p* < 0.001). Finally, there was a significant effect of trait RNT (*b* = 0.25, *SE* = 0.09, *t*(34.09) = 2.87, *p* < 0.001), indicating that those who reported a higher propensity to engage in RNT on an established self-report measure also reported more intense and frequent worries and ruminations on our survey (Fig. 3B).

Individuals with higher levels of trait RNT were those rating their idiographic worries to be more frequent and intense (Fig. 3B for RNT: PTQ *b* = 0.28, *SE* = 0.09, *t*(38.89) = 3.19, *p* = 0.003;). Additionally, trait RNT was related to more frequent and intense ruminations on our survey (*b* = 0.21, *SE* = 0.09, *t*(38.27) = 2.40, *p* = 0.021). These results highlight individuals’ idiographic worries and ruminations map onto trait measures of RNT. (For models testing trait worry and rumination measures from the start and end of the semester, see Supplemental Results, Supplemental Fig. 3, and Supplemental Table 2.)

### Idiographic worry and rumination statements elicit subjective affect and physiological arousal during imaging

The idiographic worry and rumination statements evoked affective responses (subjective feelings and autonomic arousal) during imaging. First, intensity ratings made on the survey were predictive of emotional intensity ratings made in the scanner (within person: *b* = 0.27, *SE* = 0.04, *t*(32.52) = 7.19, *p* < 0.001, and between person: *b* = 0.25, *SE* = 0.09,* t*(37.58) = 2.64, *p* = 0.012; Fig. 3C). Next, we compared the worry, rumination and neutral statements on emotional valence and intensity. Both worry (*b* =  − 0.92, *SE* = 0.03,* t*(2054.23) =  − 34.30, *p* < 0.001) and rumination (*b* =  − 1.06, *SE* = 0.03, *t*(2054.11) =  − 39.81, *p* < 0.001) statements were rated as more negative than neutral statements (Fig. 3D). Similarly, both worry (*b* = 1.08, *SE* = 0.04, *t*(2080.06) = 26.35, *p* < 0.001) and rumination (*b* = 1.42, *SE* = 0.04, *t*(2080.06) = 34.69, *p* < 0.001) statements were rated as more intense than neutral statements (Fig. 3D).

We also tested whether engaging with these personal statements evoked physiological arousal, an objective indicator of heightened affective response. A hierarchical model regressing heart rate from each trial on a categorical statement type predictor (worry, rumination, or neutral) found heart rate increased during rumination (*b* = 0.13, *SE* = 0.20, *t*(2007.01) = 0.639, *p* = 0.523) and worry (*b* = 0.27, *SE* = 0.20, *t*(2007.01) = 1.349, *p* = 0.177) compared with neutral trials, but not significantly so. However, a second model using continuous emotional intensity ratings as a predictor found that heart rate was significantly related to intensity ratings (*b* = 0.26, *SE* = 0.09,* t*(1983.00) = 3.099,* p* = 0.002; Fig. 3E), regardless of whether the statement was a worry or rumination statement. This suggests that negative emotional intensity—a shared feature of worry and rumination—may be a more potent predictor of psychophysiology than the categorical labels.

### RSA demonstrates distributed brain areas encode worry and rumination similarly

**Fig. 4:**
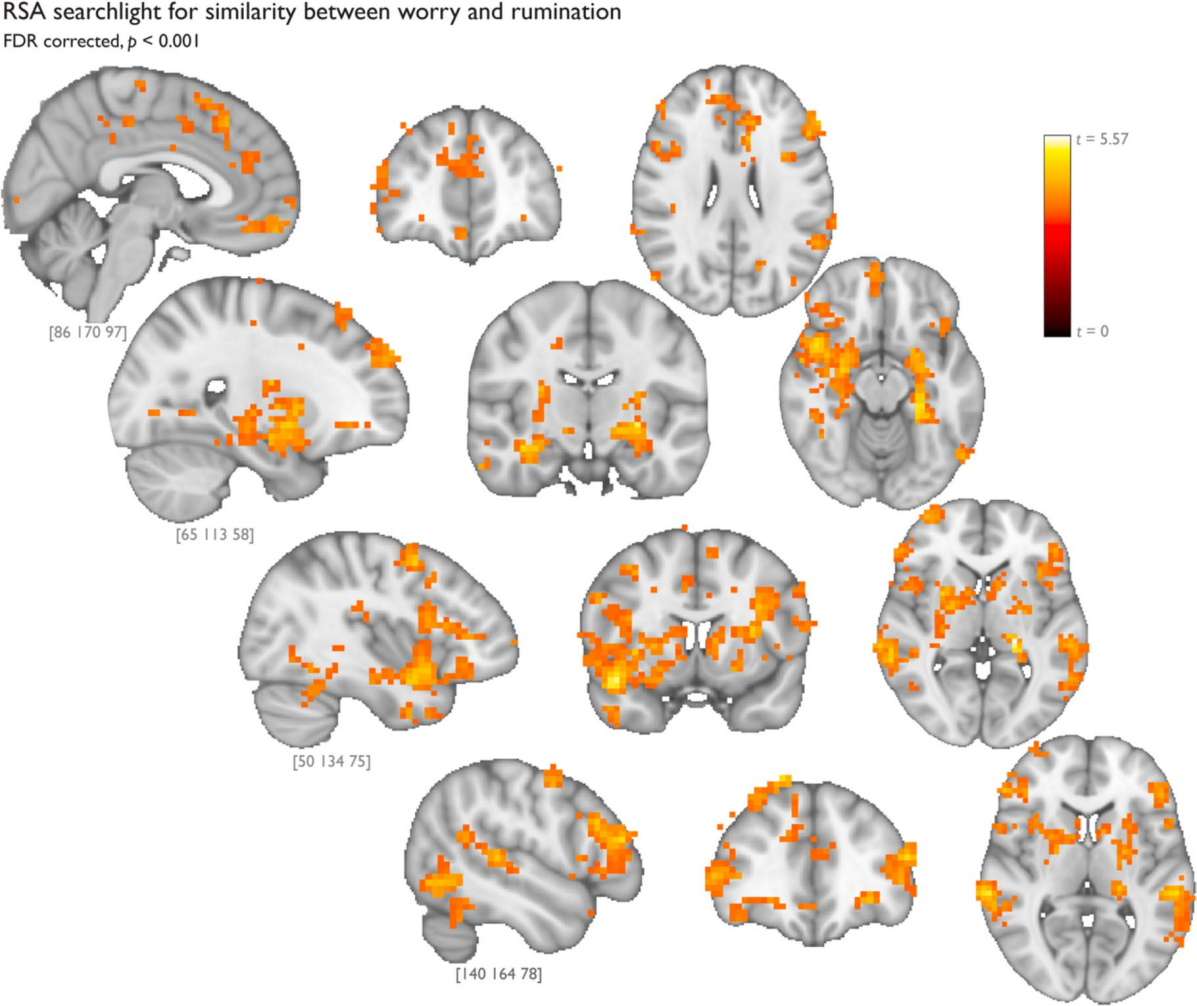
Searchlight RSA identified voxels encoding worry and rumination trials similarly to one another but different from neutral trials. This searchlight sought to identify voxels that distinguish worry and rumination from neutral. The colored bar shows the range of *t* values for statistical strength of the match between the voxel patterns and the model pattern of worry similar to rumination but dissimilar from neutral. All of the clusters shown here survive FDR correction

Our primary analysis used a searchlight to identify clusters of voxels whose activation pattern is similar for worry and rumination, but different for neutral statements. This analysis yielded clusters in nodes of the default mode, the salience, and the lateral frontoparietal networks, including the ventromedial PFC, ACC, dorsolateral PFC, insula, hippocampus, amygdala, lateral temporal lobe, and cortical midline structures. The FDR-corrected map of all clusters is displayed in Fig. 4 (also see Supplemental Table 5). In addition, we used a searchlight RSA to identify where in the brain worry and rumination are represented differently (and ignoring the neutral trials). This analysis yielded a small number of clusters in the inferior parietal lobule, lateral temporal cortex, and dorsolateral PFC. However, none survived FDR correction (see Supplemental Fig. 7 for uncorrected map).

#### Specificity analysis: searchlight RSA of IAPS images does not yield brain regions overlapping with primary worry and rumination RSA

To determine the specificity of the RNT searchlight effects and rule out the possibility that our effects were simply due to valence, we assessed whether nonidiographic and negatively valenced visual stimuli would engage similar structures in the brain. To compare the neural features distinguishing negative worries and ruminations from neutral prompts to those distinguishing negative from neutral IAPS images, we conducted a similar searchlight RSA of the IAPS viewing task. In the subset of participants who completed all trials of the IAPs paradigm (*N* = 23; see *Methods*), the model yielded clusters in the dorsomedial PFC and the right lateral occipital cortex that differentiate negative from neutral trials. However, none of the voxels survive FDR correction. When both the IAPS and RNT maps are uncorrected at *p* < 0.001, not only is the IAPS map much sparser, but there are few voxels overlapping between them (Supplemental Fig. 9). This suggests that the similarity between worry and rumination trials, compared with neutral trials, is not likely due to valence alone. Rather, more complex shared features of worry and rumination that define RNT (e.g., autobiographical, verbal, internally generated) may drive the effects from the RSA searchlight.

## Discussion

Repetitive negative thinking is a higher-order cognitive process that theoretically reflects conceptual similarities between content-specific types of thinking, such as worry and rumination. Despite this conceptual overlap, neuroscience studies have almost exclusively investigated worry and rumination separately. Therefore, we know relatively little about the degree of neural overlap between them and have sparse neurobiological evidence indicating that RNT is indeed a shared process between worry and rumination. We addressed this gap by developing a novel idiographic paradigm to quantify the behavioral, physiological, and neural similarities between worry and rumination. We employed a searchlight RSA that demonstrated worry and rumination are encoded similarly across unique worries and ruminations but not neutral prompts. Specifically, in line with theories of RNT, we identified clusters within functional brain networks implicated in self-referential thinking, memory retrieval and emotional memory, cognitive control, and salience detection (Burrows et al., 2017; Koster et al., 2011; Menon, 2011).

The identified clusters, including ventromedial PFC, ACC, insula, amygdala, hippocampus, lateral PFC, STS/angular gyrus, and others, may contribute to an “RNT system” that relies on similar structures for worry and rumination. For example, within the default mode network, the ventromedial PFC encodes the subjective, affective value of emotional stimuli (Winecoff et al., 2013) and integrates these valuations with conceptual information and schemas (Chang et al., 2021; Ghosh et al., 2014; Roy et al., 2012). During both worry and rumination, cognitions that are personal and negatively valanced, the ventromedial PFC could be coding the affective value and meaning of the thoughts in the greater context of one’s beliefs about oneself and the world.

Moreover, regions within the medial temporal lobe, including the hippocampus and amygdala, showed substantially similar activation patterns during ruminative and worry prompts (compared with the neutral prompts). The hippocampus instantiates not only autobiographical memory recall, but also simulation of future scenarios, which ultimately guides ongoing decision making (Schacter, 2012; Zielinski et al., 2020). These are core processes of both worry and rumination (Harvey et al., 2004; Heller & Bagot, 2020), which are often perceived as problem-solving strategies by those engaging in RNT. Also intimately involved in memory processes, the amygdala encodes the salience of incoming stimuli as well as previously tagged memories (LeDoux, 1996; Phelps & LeDoux, 2005; Todorov et al., 2008). Together, these regions may coordinate the retrieval and/or construction of meaningful, emotional scenarios that are often replayed during RNT.

Common representations between worry and rumination were also observed in salience network regions, including the ACC and anterior insula. Neural similarity between worry and rumination in the insula could reflect integration of similar salience signaling of perceptual sensations (i.e., increased heart rate; Demnitz-King et al., 2021) and negative cognitions and memories (Uddin, 2015). Furthermore, the ACC may be encoding the global, seemingly “catastrophic,” negative perspectives that are similar across both worry and rumination (Kalisch & Gerlicher, 2014). Finally, the lateral PFC, which supports goal-oriented executive function, may be coding a shared lack of inhibiting perseveration and diverting attention during worry and rumination (Koster et al., 2011).

The observed neural similarities between worry and rumination differed from effects seen in a similar RSA contrasting negative and neutral IAPS images. Both analyses distinguished stimuli that differed in valence (negative from neutral); yet the results from these analyses showed little to no spatial overlap, with much stronger effects in the RNT analysis. This divergence suggests that the shared neural features between worry and rumination are not likely explained simply by valence but by other stimulus features core to RNT (autobiographical, personally meaningful, evoking complex endogenous mental processes). This is in line with recent work showing that different types of aversive stimuli, such as images and sounds, exhibit a mixture of shared and unique neural features (Čeko et al., 2022). However, because the IAPS analysis included fewer participants and total trials than the RNT analysis, additional work is needed to solidify this conclusion.

We also found evidence that physiological arousal was related to negative emotional intensity, a shared feature of worry and rumination. Specifically, we found that participants’ heart rate was related to increasing subjective emotional intensity ratings (regardless of whether it was a worry, rumination, or neutral prompt). Although some previous research linked worry more tightly to heart rate (Ottaviani et al., 2016) and heart rate variability (Aldao et al., 2013), our results suggest that emotional intensity, rather than thought type, may drive the connection between RNT and heart rate. Overall, the physiological and neuroimaging evidence highlight that the shared features of between worry and rumination relate to nervous system function and provide support for RNT as a content-independent, higher-order process*.*

The current study has several limitations. First, in this sample, emotional intensity was unexpectedly related to the RNT type. Worries were rated as more intensely negative than ruminations in the survey and fMRI paradigm, which means that the reported emotion intensity effects, for example relating to heart rate and PCC amplitude, may have been more influenced by worries than ruminations. One possibility for why worry was rated more intensely negative than ruminations is because data collection occurred just before final exams. This sample of university students may have provided more proximal, salient worries about impending exams compared with ruminations about more distal past events. Future research should assess the temporal proximity and additional event features from statements. A second limitation was that unlike the worry and rumination statements, the neutral prompts were provided *to* the participants and seen in the scanner for the first time. This may have confounded familiarity between the worry/rumination and neutral conditions. We explored this by conducting an RSA excluding neutral trials to assess where worry and rumination were represented dissimilarly. Although we cannot interpret null results, the lack of significant clusters in this analysis tentatively supports their similarity regardless of the neutral trials. A third limitation was that while our paradigm used personalized stimuli to promote a more naturalistic experience of RNT, the experimental instruction and trial duration may not have evoked a naturalistic RNT process. Whereas naturally occurring worry and rumination are often spontaneous, or cued by the environment, we presented them directly to participants in the context of an fMRI task. This could have instantiated a more controlled, goal-directed process of engaging with the prompts than everyday worry and rumination, which may recruit distinct neural structures. Moreover, the generalizability of these results is constrained by the private collegiate sampling in Florida. Our sample exhibited socioeconomic (approximately 46% of participants reported belonging to a minoritized group). but additional community and clinical samples are needed to increase representation. Finally, our study included more participants and more fMRI task trials compared with similar work (Steinfurth et al., 2017); however, a formal a priori power analysis was not conducted. A sensitivity analysis performed by G*power (version 3.1.9.7; Faul et al., 2009) with our sample 39 participants, using a 0.05 error probability indicates that we have 95% power to capture moderate effect sizes (Cohen’s d = 0.46), but smaller effects might have been undetected.

In summary, this study paired a novel, idiographic worry and rumination paradigm with multivariate RSA to capture the similarity between worry and rumination—ultimately reflecting the neurobiological processes underlying RNT. We provide crucial evidence corroborating clinical and cognitive research that RNT is a shared process, involving the central and peripheral nervous system, across a wide range of specific worry and rumination thoughts. Worry and rumination exhibit shared representations across distributed brain regions from theoretically supported brain networks implicated in self-referential thinking, memory, salience detection, and cognitive control. Identifying how the brain enacts RNT is an important step toward better understanding and treating a range of psychological disorders that are intimately connected with negative cognition.

## Supplementary information

Below is the link to the electronic supplementary material.

Supplementary file1 (DOCX 8.28 MB)

## Data Availability

Https://osf.io/ax86y/.

## Code availability

https://osf.io/ax86y.
