# Supplementary material for: Worry and rumination elicit similar neural representations: neuroimaging evidence for repetitive negative thinking
Source: Cogn Affect Behav Neurosci. 2024 Nov 19;25(2):488–500. doi: 10.3758/s13415-024-01239-z (PMC11906554; doi:10.3758/s13415-024-01239-z)
Supplement: Supplementary file 1 — Supplementary file1 (DOCX 8.28 MB) [file 13415_2024_1239_MOESM1_ESM.docx]

**Supplemental Methods**

*Worry and Rumination Survey Definitions and Instructions*

For full transparency and clarification about how the participants learned the difference between worry and rumination, we have provided the text directly from the worry and rumination generation survey. Additionally, the written experimenter protocol for this portion of the laboratory session is presented. After this text, we have also provided the written experimenter protocol for the *practice* fMRI task, where participants were reminded of the worry and rumination concepts and learned how to complete the worry and rumination task they would perform in the scanner. This collection of experimental materials and protocols illustrates all of the steps that were taken to ensure that participants not only understood the difference between worry and rumination, but were able to engage with the statements meeting the definitions of worry and rumination.

Worry and Rumination Survey definitions:
The following survey will be referring to the terms**worry** and **rumination**. It is important for you to understand what these terms mean before you continue.

**Worry** refers to when we think a lot about things that we are concerned about happening*in the future.*

 - Example of a worry: "I am worried that no one will ask me to the spring dance next month"

**Rumination** refers to when we mull things over in our heads that have happened to us*in the past.
 -*Example of rumination: "I felt so disappointed last fall when no one asked me to the dance"

These different types of thoughts are often negative and may pop up without us wanting them to. They are also very common thoughts that many people experience.

*Worry and Rumination Survey Written Experimenter Protocol: “BEFORE THEY MOVE ON ASK IF THEY HAVE ANY QUESTIONS ABOUT THESE CONCEPTS: “do you have any questions about worry or rumination?” If no, say “Ok if you have any questions moving forward just let me know“*

Worry and Rumination Survey mandatory understanding check (participants were not able to continue until answering these correctly):

Here is a description of a situation in which Timothy is experiencing **worry:**
Timothy is a student-athlete who is taking a full class load. He spends a lot of time studying for classes and usually earns good grades, and he also cares about performing well on the soccer team. Timothy's coach recently told him that he needed to increase his focus and dedication to practice and games, or he might be cut from the team. Since then, Timothy has been thinking a lot about the team's upcoming soccer game and the prospect of being cut from the team if he doesn't do well.

Which of the following best characterizes Timothy's **worry**?

- "I hope that I do well on my exam next month"
- "I really wish I had not skipped practice last week."
- "I am really worried that the coach will cut me from the team."

Here is a description of a situation in which Leticia is experiencing **rumination:**
Leticia is an accountant who works hard at her job and cares about her performance. When she had to give a presentation in front of her coworkers last month, she felt very nervous, which made her voice shake and caused her to fumble over her words. Leticia has been thinking a lot lately about how embarrassing it felt to appear nervous during the presentation and stumble over her words in front of colleagues. 

Which of the following best characterizes Leticia's **rumination**?

- "I cant stop thinking about how nervous I am for this presentation next week."
- "I can't believe I looked so nervous during my presentation last month. "
- "I spent too much time preparing for my presentation last weekend"

*Worry and Rumination Survey Experimenter Protocol: “They will then move to a quiz that requires them to identify worries and ruminations. Clarify the definitions if they need it.”*

Worry and Rumination Survey example worry statement generation screen:

Here is your highest ranked topic of worry. Take a few moments to think about your worries surrounding this topic, including the thoughts that come to mind when you worry about it and why you are concerned about this topic.

- *Friends*

When you are ready, please complete the following prompts with statements that describe your worries. As a reminder, worry is focused on future events.

Your sentences should:

- **Be concise and focused on your main worry**
- **Provide enough detail** so that if you saw the sentence again you would remember exactly what you meant by it

An example of a good response is: "that my friends will exclude me from their plans to attend Jose's party."

We ask that you complete *at least four*sentences about this topic of worry. If you can generate more than four sentences, please do so. Please ask the experimenter if you have any questions about these sentences, or the term worry more generally.

| I am worried... that I will fail chemistry this semester |  |
| --- | --- |
| I am worried... |  |
| I am worried... |  |
| I am worried... |  |

Worry and Rumination Survey example rumination statement generation screen:

Here is your highest ranked topic of rumination. Take a few moments to think about your rumination surrounding this topic, including the thoughts that come to mind when you ruminate about it and why you think back on this topic.

- school

When you are ready, please complete the following prompts with statements that describe your rumination. As a reminder, rumination is focused on past events.

Your sentences should:

- **Be concise and focused on your main worry**
- **Provide enough detail** so that if you saw the sentence again you would remember exactly what you meant by it

An example of a good response is: "how upset I was when my professor called on me and I didn't know the answer"

We ask that you complete *at least four* sentences about this topic of rumination. If you can generate more than four sentences, please do so. Please ask the experimenter if you have any questions about these sentences, or the term rumination more generally.

I often think back on... how bad it was that I wasn't prepared for class when I should have been

I often think back on...

I often think back on...

I often think back on...

Worry and Rumination fMRI task written protocol, completed by either the first or second author approximately one week following the Worry and Rumination Generation Survey completion:

“*Scan Session: Worry and Rumination fMRI Task Practice Experimenter Protocol:*

***Worry/rumination definition refresher***

- 1. *Tell the participant, we’re now going to practice the task that you’ll do later in the*

*MRI scanner so we can answer any questions and make sure you understand the*

*instructions.*

- 1. *Say, we first want to remind you about the difference between worry and rumination, as this will be important for the task you do today.*
  2. *Worry and rumination both involve negative thoughts that pop up repeatedly, usually about ourselves or the situations in our lives. The main difference is that worry is focused on the* ***future****, or things that haven’t happened yet, whereas rumination is about the* ***past****.*
  3. *If I say, “I can’t stop thinking about a difficult exam coming up next week,” would that be an example of worry or rumination?  Elicit that this is worry.*
  4. *If I say, “I’ve been thinking a lot about past exams I did poorly on,” would that be an example of worry or rumination?  Elicit that this is rumination.*

***Review of participant-generated worry and rumination statements***

1. *Say, While you’re doing the MRI part of the study, we’re going to ask you to think about some of the worry and rumination statements you came up with the other day, as well as some additional topics. Before we explain and practice the task, we’re going to remind you about the statements you came up with.*
2. *Give the participants the printout of their generated worry and rumination statements.*
3. *Tell the participant, please read over this list of statements you came up with. As a reminder, these were statements about things you worry about or ruminate on. Make sure you can clearly remember what each of the statements is referring to because you won’t have much time during the task to try to recall what you meant.*
4. *When the participant is finished reading through, confirm that they remember all of the statements.*
5. *Also ask them if any of the worries have passed temporally, for example if people wrote a worry statement about their “exam next week”, has that exam already passed?*

- *For the statements that are no longer future oriented, ask if they have a very similar worry regarding that topic that is still future-focused.*
- *Make note of this on the checklist & update the computer task file*

***Practice task introduction***

1. *Say, Now we’re going to practice the task that we’ll have you do while you’re in the MRI machine.*
2. *During this task, you’ll see a bunch of different statements come up on the screen. These statements will include a mix of the worry and rumination statements you came up with during your last session, as well as some other statements we added.*
3. *When a statement appears on the screen, we would like for you to worry or ruminate about this statement as you normally would for as long as it remains on the screen. You’ll know to worry if the statement starts with “ I am worried…”, or to ruminate if it starts with “I often think back on”. If it’s not a worry or rumination statement, it will say “I sometimes think…” so you should reflect on the content of the statement just like the others.*
4. *The statements are not displayed for a very long time so please try to engage with the statement as much as possible.*
5. *When the statement goes away, you’ll be asked to your reaction to the statement. Specifically, you will indicate if you were feeling good, bad, or neither good nor bad. Then, you will rate how strong or intense your feelings were while thinking about the statement.*
6. *We want this to feel just like it would when you think hard about these topics in during your day-to-day life. So, when a statement appears, think about how you would normally worry or ruminate on the topic and do just that. Mull the statement over in your mind and immerse yourself in that thought.*
7. *Be sure that if it is a rumination statement, you think back on the topic; that if it is a worry statement, you think about this happening in the future. If it is a statement you did not create (that is, one that we provided), just think about that statement.*

***After Task Practice***

1. *Have the participant complete the task using the button box and answer any questions that come up.*
2. *Ask, Do you have any questions? Were you able to ‘get into’ all of the statements?*
3. *Provide instructions: During the task, you’ll see different statements than the ones we just showed you, but they will also come from the list of statements you provided, combined with a few more that we’ve added in.*
4. *Tell the participant, We’re now going to get you set up to complete the MRI task.*
5. *Take the person to the scanner waiting room and have them take a seat. Say, Please wait here while I prepare a few things with our scanner technician, who will help us get set up.*

*Validation of participant statements as worry or rumination*

We tested whether participants wrote clearly differentiated worry and rumination statements by training a large language model to classify the 24 worry and 24 rumination statements for each participant (total *N*=2078 statements) based on the exact instructions we provided to participants (see *Worry and Rumination Survey definitions* section above). The Mistral-7B-Instruct-v0.2 Large Language Model (https://mistral.ai/) was provided 2 worry and 2 rumination examples from the survey instructions above and asked to “classify whether a statement is WORRY or RUMINATION”.

To optimize model performance, we provided additional structure by requesting that the output be, “specific in the breakdown, giving direct examples from the statement to justify the answer.” We provided examples of the output we expected, for example for the worry “that the coach will cut me from the team”, we provided this example breakdown: "Let’s break the statement down step by step. The act of being removed from the team has yet to occur, making this a future statement. Additionally, being removed from the team could be a negative experience for the speaker of the statement. Therefore, this statement is future-focused on an undesirable outcome, meeting the definition of worry."

The outcome metrics from the model are precision, recall and an ‘F-1’ score. Precision refers to the percent of statements classified by the model as worry, for example, were indeed worry. Recall refers to the percent of all true worries given to the model that were correctly labelled as such by the model. Because the model may provide “neither” or “other” labels if/when a determination is not made, the precision and recall values are distinct. Finally, the F-1 score is the harmonic mean of precision and recall; it balances precision and recall by multiplying them, dividing by their sum, and then multiplying by 2. Each of these metrics were determined for worry and also for rumination.

*fMRI Data Preprocessing*

Anatomical data preprocessing. The T1-weighted (T1w) image was corrected for intensity non-uniformity (INU) with N4BiasFieldCorrection (Tustison et al. 2010), distributed with ANTs 2.2.0 (Avants et al. 2008, RRID:SCR_004757), and used as T1w-reference throughout the workflow. The T1w-reference was then skull-stripped with a Nipype implementation of the antsBrainExtraction.sh workflow (from ANTs), using OASIS30ANTs as target template. Brain tissue segmentation of cerebrospinal fluid (CSF), white-matter (WM) and gray-matter (GM) was performed on the brain-extracted T1w using fast (FSL 5.0.9, RRID:SCR_002823, Zhang, Brady, and Smith 2001). Brain surfaces were reconstructed using recon-all (FreeSurfer 6.0.1, RRID:SCR_001847, Dale, Fischl, and Sereno 1999), and the brain mask estimated previously was refined with a custom variation of the method to reconcile ANTs-derived and FreeSurfer-derived segmentations of the cortical gray-matter of Mindboggle (RRID:SCR_002438, Klein et al. 2017). Volume-based spatial normalization to one standard space (MNI152NLin2009cAsym) was performed through nonlinear registration with antsRegistration (ANTs 2.2.0), using brain-extracted versions of both T1w reference and the T1w template. The following template was selected for spatial normalization: ICBM 152 Nonlinear Asymmetrical template version 2009c [Fonov et al. (2009), RRID:SCR_008796; TemplateFlow ID: MNI152NLin2009cAsym].

Functional data preprocessing. For each of the 5 BOLD runs found per subject (across all tasks and sessions), the following preprocessing was performed. First, a reference volume and its skull-stripped version were generated using a custom methodology of fMRIPrep. The BOLD reference was then co-registered to the T1w reference using bbregister (FreeSurfer) which implements boundary-based registration (Greve and Fischl, 2009). Co-registration was configured with nine degrees of freedom to account for distortions remaining in the BOLD reference. Head-motion parameters with respect to the BOLD reference (transformation matrices, and six corresponding rotation and translation parameters) are estimated before any spatiotemporal filtering using mcflirt (FSL 5.0.9, Jenkinson et al., 2002). The BOLD time-series, were resampled to surfaces on the following spaces: fsaverage5. The BOLD time-series were resampled onto their original, native space by applying a single, composite transform to correct for head-motion and susceptibility distortions. These resampled BOLD time-series will be referred to as preprocessed BOLD in original space, or just preprocessed BOLD. The BOLD time-series were resampled into standard space, generating a preprocessed BOLD run in [‘MNI152NLin2009cAsym’] space. First, a reference volume and its skull-stripped version were generated using a custom methodology of fMRIPrep. The fMRIPrep pipeline extracts several confounding time-series, including framewise displacement (FD), DVARS, three region-wise global signals extracted within the CSF, the WM, and the whole-brain masks, as well as a set of physiological regressors for noise correction (CompCor, Behzadi et al., 2007). However, these confounds were not included in the first level models in this study.

Head-motion estimates calculated in the correction step were also placed within the corresponding confounds file. The confound time series derived from head motion estimates and global signals were expanded with the inclusion of temporal derivatives and quadratic terms for each (Satterthwaite et al., 2013), however only the 6 primary motion regressors were used for the first level analyses. Frames that exceeded a threshold of 0.5 mm FD or 1.5 standardized DVARS were annotated as motion outliers. All resamplings can be performed with a single interpolation step by composing all the pertinent transformations (i.e., head-motion transform matrices, susceptibility distortion correction when available, and co-registrations to anatomical and output spaces). Gridded (volumetric) resamplings were performed using antsApplyTransforms (ANTs), configured with Lanczos interpolation to minimize the smoothing effects of other kernels (Lanczos, 1964). Non-gridded (surface) resamplings were performed using mri_vol2surf (FreeSurfer).

*Univariate Models*

For the univariate GLM contrasts, the preprocessed data were input to AFNI’s 3dDeconvolve function, which concatenated the 5 functional runs and estimated the model with task and nuisance regressors. There were 6 task regressors: 1 for each of the worry, rumination and neutral condition statement displays and 1 for each of the worry, rumination and neutral rating periods. These predictors were fit using ‘GAM’, a gamma function with one parameter for the peak. In addition to these regressors-of-interest, scanner drift and 6 standard motion regressors were also included. This model yielded in a single beta coefficient for each voxel, for each condition. Condition contrasts were then specified: worry – rumination, and finally worry + rumination – neutral (reflecting RNT). Then, for each of these contrasts, we used 3dMEMA to conduct a mixed effects group analysis, using the beta coefficient and t-value for each voxel of each subjects’ contrast maps. For both group analyses, we used 3dClustsim to estimate the minimum cluster size with an *a* = .05 and *p* = 0.001. This threshold was then applied to create the final univariate contrast maps.

The final univariate GLM we tested was an amplitude modulation analysis to assess brain activity that varied with participants’ intensity ratings. The purpose of this analysis is to determine which brain regions covary with emotional intensity, a shared feature of RNT, regardless of statement type (worry or rumination). To this end we used the “amplitude modulation” tag in AFNI’s 3dDeconvolve to modulate trial-by-trial brain activity by the emotional intensity rating given on that trial. The same ‘GAM’ HRF shape and motion regressors used for the above univariate models were also used for this analysis. Additionally, the same cluster size correction procedure described above was also used to threshold the amplitude modulation output map.

**Supplemental Results**

*The worry and rumination survey statements*

The average intensity and frequency ratings were calculated for each of the worry and rumination topics. The heatmaps in Supplemental Figure 1 display these averages, with each row representing a subject and each column representing what rank the subject had given to the topic.

We also demonstrate that, given the instructions provided to participants, a large language model can accurately classify their written statements as either worry or rumination. The model achieved 93% precision in classifying both worry and rumination statements, meaning worry statements were not likely to be classified as rumination or vise-a-versa (Supplemental Figure 2). The model recall, or the percent of all inputted worry statements that were correctly classified as worry (not *neither* or *other)* was 86%. For rumination, model recall was lower at 75%. The F-1 score, which incorporates the model’s precision and recall, was 89% for worry and 83% for rumination. These model results, in conjunction with our detailed instructions, strongly suggest participants were indeed able to differentiate between worry and rumination. Examples of correctly and incorrectly classified worry and rumination statements as well as the model rationale for the classification is provided in Supplemental Table 7).

Still, the model precision and recall results indicate that a portion of statements were *not* correctly identified. This imperfect classification may be, in part, due to model missing the key context and autobiographical knowledge that participants had. One contextual factor was that each statement written by participants began with a stem identifying it as worry or rumination, such as “I am worried…”. The stems had to be removed to not bias the model, but were necessary for understanding for some participant statements. For example, the statement, “I am being used,” is much less ambiguous when it is paired with the “I am worried that…” stem. Similarly, the rumination statement “when I do not talk enough at a social gathering,” may be less obvious to the model compared to the participant who wrote it following, “I often think back on…”. It is likely that a portion of statements were not correctly categorized because the original statements were dependent on the stems.

Another reason for imperfect classification is that only participants have intimate knowledge of the personal events and beliefs that motivated their statements. Although instructed to be specific, some participants wrote relatively vague statements that allude to specific scenarios that they had in mind. For example, a rumination about “my fake relationships,” refers to specific experiences that are known to the participant but not explicitly described. This statement, and others like it, was classified as “neither”, with the model breakdown stating, “*This statement is not about a specific event in the past or future. Instead, it is a reflection on the concept of having fake relationships. While this thought may bring up negative emotions, it is not focused on a past or future occurrence. Therefore, this statement does not fit neatly into the definitions of either worry or rumination. It could be considered a more generalized form of negative thought or introspection.*” This breakdown illustrates both the strengths and the weaknesses of the language model. Considering the autobiographical and contextual knowledge that participants had, it is likely that this model underestimated how well participants identified and differentiated worry and rumination in the scanner.
 A data frame containing the full data, including the original statement and label (worry or rumination) as well as the model’s label and reasoning, is provided at https://osf.io/ax86y/

*Associations between the survey and trait measures from both beginning and end of semester*

In the main text, we reported that more intense and frequent worries on our survey were related to higher trait levels of RNT. We also examined trait worry and rumination, measured by the Penn State Worry Questionnaire (PSWQ; Meyer et al., 1990) and the Ruminative Response Scale (RRS; Nolen-Hoeksema & Morrow, 1991) respectively, which were both positively related to statement intensity and frequency on our survey (PSWQ *b* = 0.35, *se* = 0.09, *t*(38.31) = 4.00, *p* < 0.001; RRS *b* = 0.23, *se* = 0.10, *t*(37.22) = 2.43, *p* = 0.020). We also found that trait RNT was related to more frequent and intense ruminations on our survey (*b* = 0.21, *se* = 0.09, *t*(38.27) = 2.40, *p* = 0.021) but not trait worry PSWQ *b* = 0.15, *se* = 0.09, *t*(39.57) = 1.69, *p* = 0.099, or trait rumination (RRS *b* = 0.06, *se* = 0.10, *t*(37.22) = 2.43, *p* = 0.020). This pattern of results highlights that our worry and rumination generation survey aligns with existing trait measures of RNT, worry, and rumination; however, these relationships are more robust for worry than rumination (Supplemental Figure 3 and Supplemental Table 2).

In addition to assessing the relationship between the worry and rumination generation survey and trait measures from time 2, we also assessed start of semester measures of RNT, worry, and rumination. At time 1, only trait rumination (measured via the RRS) was positively related to worry statement ratings on our survey (*b* = 0.20, *se* = 0.09, *t*(39.05) = 2.13, *p* = 0.039).

We also tested whether trait RNT, worry, and rumination changed between time 1 and 2. RNT decreased on average (M_difference_ = -4.97, *t =* -3.36 *, p =* 0.002) from the start to the end of the semester, as did rumination (M_difference_ = -5.79, *t =* -2.87 *, p =* 0.007). However, worry increased on average, although this difference was slightly above the threshold for statistical significance (M_difference_ = 3.44, *t =* 1.99 *, p =* 0.055). As discussed in the main text, this increase in worry only may reflect the saliency and proximity of final exams at the end of semester.

*The relationship between heart rate variability and fMRI task statements*

Although heart rate variability was not our primary physiological metric on interest (due to the short time window for each trial) it is a common metric used in the affective and clinical science field. Therefore, we include it here in the supplement for completeness.

A hierarchical model using heart rate variability as the outcome showed a very similar pattern of results to the model presented in the main text with average heart rate across the trial as the outcome. Specifically, heart rate variability from each trial was lower during worry compared to neutral trials, but not significantly so (*b* = -0.073, *se* = 0.04, *t*(1932) = -1.70, *p* = 0.089). Heart rate variability was also lower during worry compared to rumination trials (*b* = -0.078, *se* = 0.04, *t*(1932) = -1.95, *p* = 0.0513). However, like the model from the main text, heart rate variability was more robustly related to continuous emotional intensity ratings (*b* = -0.04, *se* = 0.02, *t*(1914.16) = -2.34, *p* = 0.019), again suggesting that the physiological responses are linked more tightly with a shared feature between worry and rumination – emotional intensity – then the thought categories themselves.

*Univariate GLM shows similar activation for worry and rumination*

We used a general linear model to determine whole-brain activation related to the worry and rumination conditions. Specifically, we examined an “RNT contrast” which compared the worry and rumination trials together and against the neutral trials. Corrected clusters (*p* = 0.001, *a* = 0.05) and their coordinates are listed in Supplemental Table 3 and displayed in Supplemental Figure 4. The RNT contrast yielded a positive cluster in the PCC/precuneus area and negative clusters in the bilateral posterior parietal cortex. Additional clusters including positive clusters throughout the medial PFC of the default mode network and negative clusters across the lateral PFC of the lateral frontoparietal control network did not survive correction.

Additionally, we directly contrasted worry and rumination to test whether there were unique neural structures that distinguished these processes. No clusters survived thresholding and cluster size correction, suggesting that worry and rumination neurally more similar than different. Prior to thresholding and correction, clusters in the PCC and the dorsolateral PFC were observed (see Supplemental Figure 5 for the uncorrected map from this contrast).

To further explore the neural commonalities of worry and rumination, we explored where in the brain a shared feature of worry and rumination – negative emotional intensity – could be tracked. We tested trial wise ratings of emotional intensity as a predictor of brain activity using a whole-brain amplitude modulated GLM. This analysis identified voxels where activity increased relative to statement intensity ratings. This model showed a pattern of activation similar to the RNT contrast, with a significant positive PCC cluster, indicating activation in the PCC is greater on trials where participants endorse a greater emotional intensity (Supplemental Figure 6, Supplemental Table 3).

*ROI-based RSA of the PCC*

The PCC was the most prominent cluster across the univariate analyses. However, few voxels in the PCC were significant in the searchlight RSA for worry and rumination. In an exploratory analysis, we determined whether the *size* of the searchlight might have impacted the results. Specifically, we tested whether the data similarity matrix across voxels in the PCC cluster from the univariate analysis (161 voxels) was significantly related to the RNT model similarity matrix (worry and rumination are similar to each other but different from neutral). In other words, rather than using a searchlight to find clusters that demonstrate our model pattern, we asked whether the PCC cluster, specifically, demonstrated our model RNT pattern. We found that the PCC ROI indeed showed a significant relationship to the model matrix (*r* = 0.079, *p* = 0.002). This suggests that the univariate and multivariate results are not in contradiction to once another regarding the PCC.

As a final step, we aimed to confirm that this ROI-based approach was indeed capturing a match between the data and model matrices by examining an ROI of no interest. Specifically, we tested an ROI of similar size (144 voxels) but a region not in the univariate RNT map and not hypothesized to be related to RNT. Specifically, we extracted voxels from the cerebellum (Vermis VIIa) and did not find that the data similarity matrix matched the model RNT matrix (*r* = 0.003, *p* = 0.908).

*Univariate IAPS contrast*

Similar to the RNT paradigm, we also derived the univariate contrast of the negative and neutral images in the IAPS task. This was done to determine whether our image viewing task successfully elicited activity in the expected regions. Confirming task activation is particularly important given that the searchlight RSA of the IAPS task did not yield clusters that survived FDR correction. The resulting negative-neutral contrast map illustrates that the IAPS task yielded significant clusters in regions associated with aversive images, including the amygdala, dorsomedial PFC, and lateral occipital lobe (Supplemental Figure 8, Supplemental Table 4). Given these significant clusters, we can be reasonably confident that the task evoked the expected effects and that the RSA not yielding clusters was not likely due to the task “not working”. However, it is certainly still possible the that the null RSA results is related to the smaller number of subjects and trials.

**Supplemental References**
Avants, B. B., Epstein, C. L., Grossman, M., & Gee, J. C. (2008). Symmetric diffeomorphic image registration with cross-correlation: evaluating automated labeling of elderly and neurodegenerative brain. Medical Image Analysis, 12(1), 26–41.

Behzadi, Y., Restom, K., Liau, J., & Liu, T. T. (2007). A component based noise correction method (CompCor) for BOLD and perfusion based fMRI. NeuroImage, 90–101.

Cox, R. W. (1996). AFNI: software for analysis and visualization of functional magnetic resonance neuroimages. Computerized Medical Imaging and Graphics, 29(3), 162–173.

Dale, A., Fischl, B., & Sereno, M. I. (1999). Cortical Surface-Based Analysis: I. Segmentation and Surface Reconstruction. NeuroImage, 9(2), 179–194.

Desikan, R. S., Ségonne, F., Fischl, B., Quinn, B. T., Dickerson, B. C., Blacker, D., ... & Killiany, R. J. (2006). An automated labeling system for subdividing the human cerebral cortex on MRI scans into gyral based regions of interest. NeuroImage, 31(3), 968–980.

Fonov, V. S., Evans, A. C., McKinstry, R. C., Almli, C. R., & Collins, D. L. (2009). Unbiased nonlinear average age-appropriate brain templates from birth to adulthood. NeuroImage, 47, S102.

Greve, D. N., & Fischl, B. (2009). Accurate and robust brain image alignment using boundary-based registration. NeuroImage, 48(1), 63–72.

Jenkinson, M. (2003). Fast, automated, N-dimensional phase-unwrapping algorithm. Magnetic Resonance in Medicine, 49(1), 193–197.

Jenkinson, M., Bannister, P., Brady, M., & Smith, S. (2002). Improved optimization for the robust and accurate linear registration and motion correction of brain images. NeuroImage, 17(2), 825–841.

Meyer, T. J., Miller, M. L., Metzger, R. L., & Borkovec, T. D. (1990). Development and validation of the Penn State Worry Questionnaire. Behaviour Research and Therapy, 28(6), 487–495.

Nolen-Hoeksema, S., & Morrow, J. (1991). A prospective study of depression and posttraumatic stress symptoms after a natural disaster: The 1989 Loma Prieta earthquake. Journal of Personality and Social Psychology, 61(1), 115–121.

Power, J. D., Mitra, A., Laumann, T. O., Snyder, A. Z., Schlaggar, B. L., & Petersen, S. E. (2013). Methods to detect, characterize, and remove motion artifact in resting state fMRI. NeuroImage, 84, 320–341.

Pruim, R. H. R., Mennes, M., van Rooij, D., Llera, A., Buitelaar, J. K., & Beckmann, C. F. (2015). ICA-AROMA: A robust ICA-based strategy for removing motion artifacts from fMRI data. NeuroImage, 112, 267–277.

Satterthwaite, T. D., Elliott, M. A., Gerraty, R. T., Ruparel, K., Loughead, J., Calkins, M. E., ... & Wolf, D. H. (2013). An improved framework for confound regression and filtering for control of motion artifact in the preprocessing of resting-state functional connectivity data. NeuroImage, 64, 240–256

Treiber, J. M., White, N. S., Steed, T. C., Bartsch, H., Holland, D., Farid, N., McDonald, C. R., Carter, B. S., Dale, A. M., & Chen, C. C. (2016). Characterization and correction of geometric distortions in 814 diffusion weighted images. PLOS One, 11(3), e0152472.

Tustison, N. J., Avants, B. B., Cook, P. A., Zheng, Y., Egan, A., Yushkevich, P. A., & Gee, J. C. (2010). N4ITK: improved N3 bias correction. IEEE Transactions on Medical Imaging, 29(6), 1310–1320.

Zhang, Y., Brady, M., & Smith, S. (2001). Segmentation of brain MR images through a hidden Markov random field model and the expectation-maximization algorithm. IEEE Transactions on Medical Imaging, 20(1), 45–57.

**Supplemental Figures**


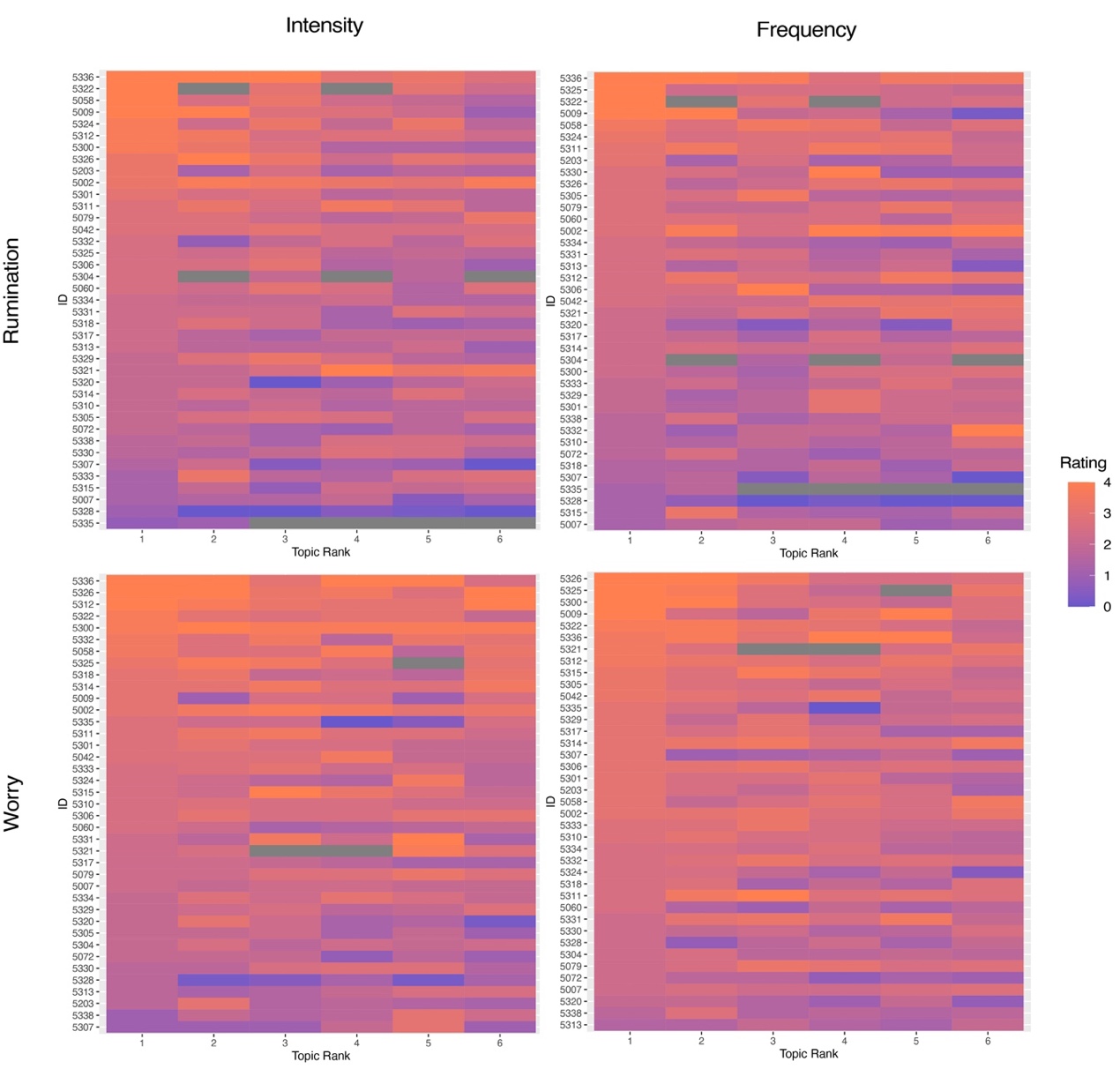


Supplemental Figure 1. Average worry and rumination statement intensity and frequency ratings by topic rank in the generation survey

*Each row represents one participant and the columns along the x-axis correspond to the topic rank (1-6). IDs along the y-axis are sorted from high to low ratings of topic 1 for visualization purposes.*


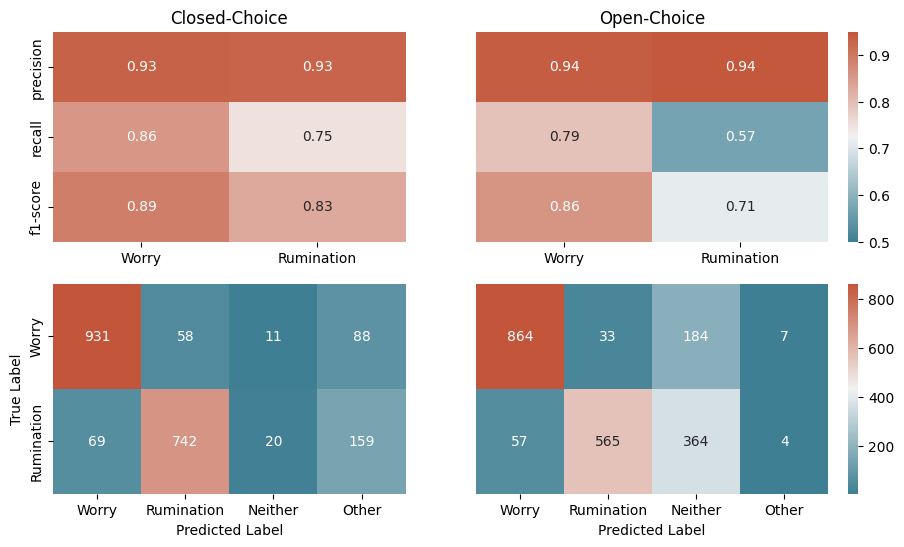


Supplemental Figure 2. Performance metrics for the large language model classifying worry and rumination statements.

*Closed choice refers to the model instructions to choose either worry or rumination. Performance metrics include precision, the percent of model-classified statements that were correct; recall, the percent of all input statements from one category that were classified correctly by the model; and f1, the harmonic mean of precision and recall. Darker shades of orange reflect better model performance.*

Supplemental Figure 3. Relationships between rumination and worry statement ratings and scores on trait rumination, worry, and repetitive negative thinking questionnaires at the start and end of the semester.

*Results from a series of multilevel regression models with survey rating as the outcome predicted by an RNT type by trait measure interaction. Significant slopes are shown with the corresponding beta and p value. Nonsignificant beta and p values are not displayed but can be found in Supplemental Table 2. Dotted lines reflect time 1 slopes and solid lines reflect time 2 slopes. Green = RNT, blue = worry, and purple = rumination.*

Supplemental Figure 4. Corrected and uncorrected univariate maps of the RNT contrast (worry + rumination versus neutral)

*Statistical maps showing t values from the mixed effects models showing at the group level where in the brain activity is greater (red) or lower (blue) for RNT (+0.5 rumination and +0.5 worry) compared to neutral trials.* ***A****) unthresholded activation map.* ***B****) thresholded at p < 0.001 and only clusters that meet the minimum cluster size from meet the 3dClustSim with an a = .05 and p = 0.001.* ***C****) Violin plots showing the distribution of each participant’s beta value (circle), per condition, in the three significant clusters from the group map. Group mean of the beta value is shown with a triangle. L. = left. R. = right.*

*PCC = posterior cingulate cortex.*

Supplemental Figure 5. Uncorrected univariate maps of the worry versus rumination contrast.

*A statistical map of t values from the mixed effects model showing at the group level where in the brain activity is greater (red) or lower (blue) for worry compared to rumination trials. No clusters met the minimum cluster size from meet the 3dClustSim with an a = .05 and p = 0.001.*

Supplemental Figure 6. Corrected and uncorrected univariate maps of the amplitude modulation by emotional intensity ratings

*A statistical map of t values from the mixed effects model showing at the group level where in the brain activity increases (red) or decreases (blue) as trial-by-trial emotional intensity ratings increase.* ***A****) unthresholded activation map.* ***B****) thresholded at p < 0.001 and only clusters that meet the minimum cluster size from meet the 3dClustSim with an a = .05 and p = 0.001.****C****) Violin plot showing the distribution of each participant’s beta value (circle) in the significant PCC cluster from the group map. Group mean of the beta value is shown with a triangle. PCC = posterior cingulate cortex.*


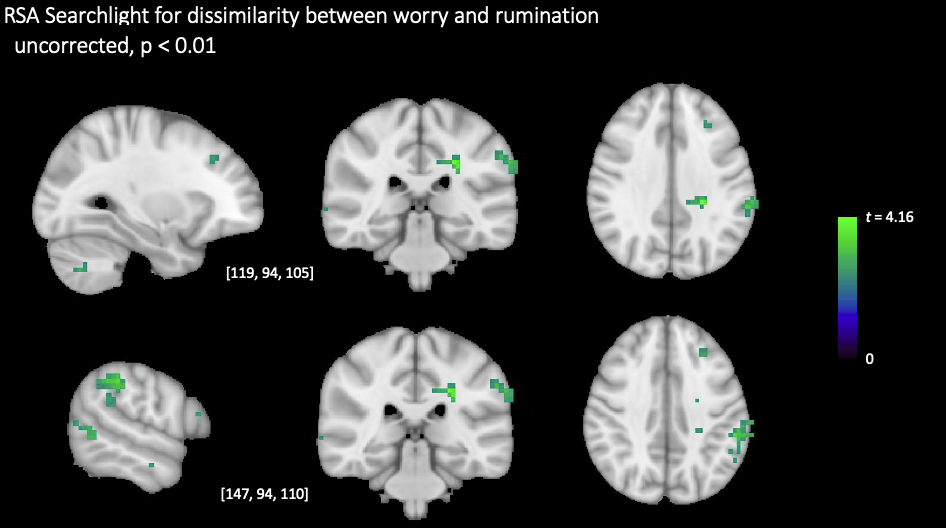


Supplemental Figure 7. Voxels encoding worry and rumination trials dissimilarly to one another according to RSA searchlight.

*This searchlight sought to identify voxels that distinguish worry from rumination; neutral trials were not included in this model. The colored bar shows the range of t values for statistical strength of the match between the voxel patterns and the model pattern of worry dissimilar from rumination. None of the clusters shown here survive FDR correction.*


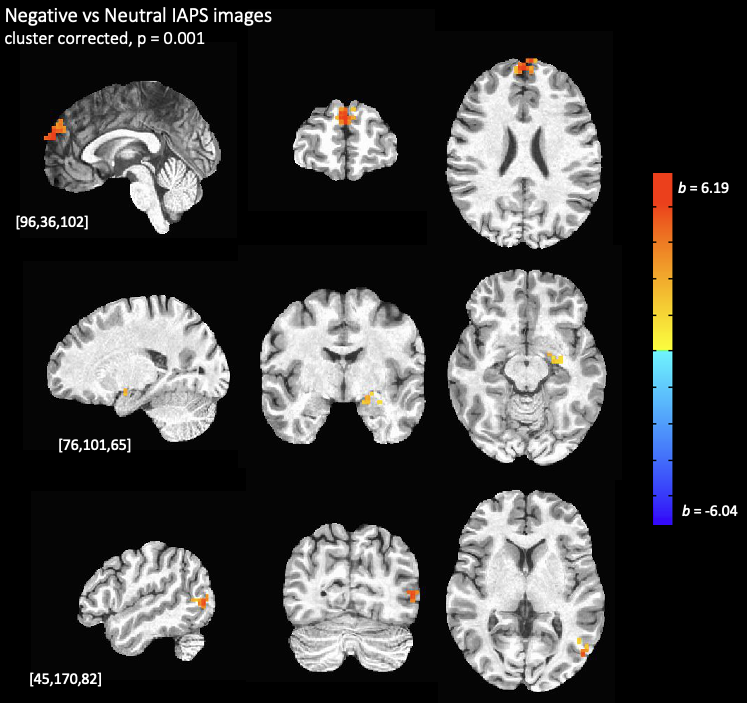


Supplemental Figure 8. Corrected univariate map of the IAPS negative-neutral contrast

*A statistical map of t values from the mixed effects model showing at the group level where in the brain activity increases (red) or decreases (blue) as trial-by-trial emotional intensity ratings increase. Only clusters that meet the minimum cluster size from 3dClustSim with an a = .05 and p = 0.001 are displayed.*


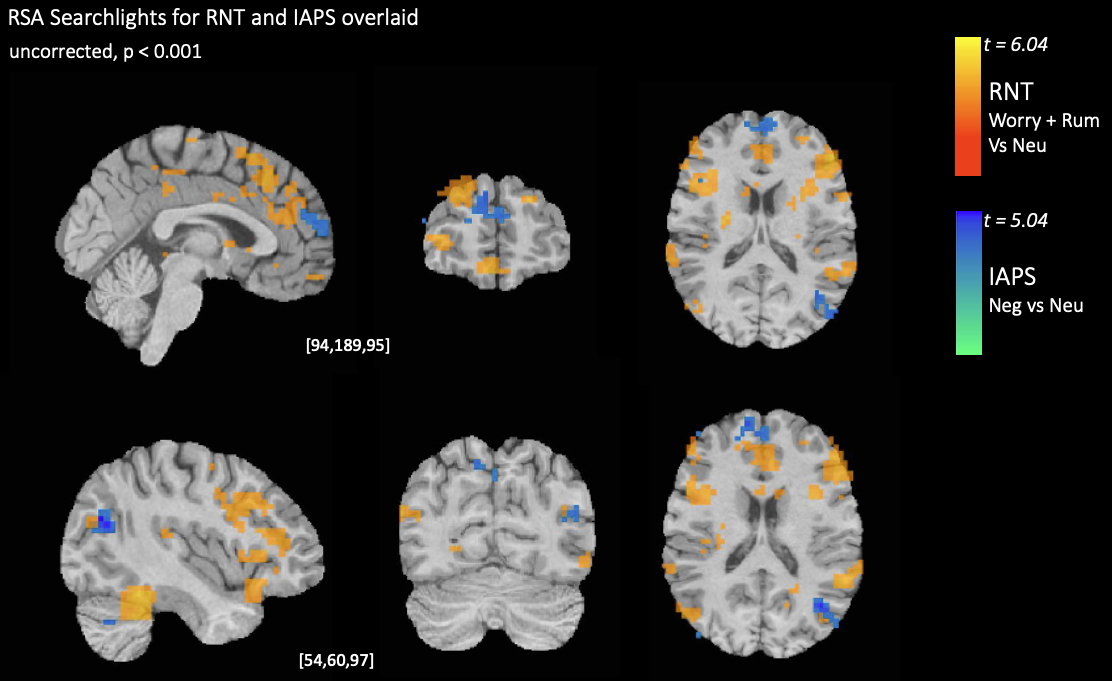


Supplemental Figure 9. Overlaying uncorrected maps from the RNT and IAPs searchlights

*Statistical map of t values for group-level searchlight RSAs for the RNT paradigm and the IAPS paradigm. Both searchlights sought to identify voxels that distinguish negative from neutral stimuli, though the stimuli features differed between the paradigms. The colored bar shows the range of t values for statistical strength of the match between the voxel patterns and the model pattern. Clusters from the IAPS analysis did not survive FDR correction, so in order to visualize their overlap, both maps were thresholded at p=0.001 with no other corrections.*

**Supplemental Tables**

Supplemental Table 1. Descriptive Statistics for trait RNT and symptom measures at time 1 (beginning) and 2 (end of semester)

|  |  | **Scanned  Participants  (N=39)** | | | **Non-scanned Participants**  **(N=75)** | |  |  |
| --- | --- | --- | --- | --- | --- | --- | --- | --- |
|  |  | **Mean** | **SD** | **Mean** | | **SD** | **Difference p-value** | |
| **Sex** |  | .41 |  | .43 | |  | .890 | |
| **Age** |  | 18.8 | .84 | 18.5 | | 1.0 | .117 | |
|  | **Range** |  |  |  | |  |  | |
| **Time 1** |  |  |  |  | |  |  | |
| RNT (PTQ) | 2 - 60 | 31.6 | 8.1 | 26.9 | | 12.3 | .023 | |
| Rumination (RRS) | 22 - 81 | 48.4 | 14.4 | 46.0 | | 16.3 | .562 | |
| Worry (PSWQ) | 25 - 76 | 49.6 | 12.0 | 51.4 | | 13.3 | .422 | |
| Anxiety symptoms (GAD7) | 0 - 21 | 7.2 | 4.9 | 7.2 | | 5.3 | .945 | |
| Depression Symptoms (PHQ9) | 0 - 22 | 6.7 | 5.4 | 6.6 | | 5.3 | .972 | |
|  |  |  |  |  | |  |  | |
| **Time 2** |  |  |  |  | |  |  | |
| RNT (PTQ) | 0 - 55 | 26.3 | 9.8 | 24.6 | | 14.5 | .467 | |
| Rumination (RRS) | 22 - 85 | 41.8 | 12.6 | 45.6 | | 17 | .184 | |
| Worry (PSWQ) | 18 - 79 | 52.4 | 12.7 | 50.5 | | 14.7 | .479 | |
| Anxiety symptoms (GAD7) | 0 - 21 | 7.1 | 5.1 | 7.1 | | 5.4 | .965 | |
| Depression Symptoms (PHQ9 | 0 - 26 | 7.7 | 5.3 | 8.0 | | 6.1 | .778 | |

*Difference p-value refers to the significance value from a two-sample t-test comparing the mean of the scanned and non-scanned participants.*

Supplemental Table 2. Multilevel linear models of survey statement ratings regressed on trait measures of RNT, worry, and rumination

|  |  | **Start of Semester** | | | | | | | | | | | |
| --- | --- | --- | --- | --- | --- | --- | --- | --- | --- | --- | --- | --- | --- |
|  |  | RNT (PTQ) | | | | Worry (PWSQ) | | | | Rumination (RRS) | | | |
|  |  | *b* | *se* | *t* | *p* | *b* | *se* | *t* | *p* | *b* | *se* | *t* | *p* |
| **Main effects** |  |  |  |  |  |  |  |  |  |  |  |  |  |
| Trait Measure |  | 0.08 | 0.10 | 0.83 | 0.414 | 0.14 | 0.09 | 1.53 | 0.133 | 0.16 | 0.09 | 1.68 | 0.100 |
| RNT Type |  | **0.28** | **0.04** | **6.37** | **< 0.001** | **0.28** | **0.04** | **6.35** | **< 0.001** | **0.28** | **0.04** | **6.38** | **< 0.001** |
| Trait Measures x RNT Type | | 0.10 | 0.04 | 2.26 | 0.024 | 0.02 | 0.04 | 0.52 | 0.601 | 0.04 | 0.04 | 0.98 | 0.33 |
| **Simple slopes** |  |  |  |  |  |  |  |  |  |  |  |  |  |
| Rumination ~ Trait Measure | | 0.08 | 0.10 | 0.83 | 0.414 | 0.14 | 0.09 | 1.53 | 0.133 | 0.16 | 0.09 | 1.68 | 0.100 |
| Worry ~  Trait Measure | | 0.18 | 0.10 | 1.85 | 0.072 | 0.17 | 0.09 | 1.78 | 0.083 | **0.20** | **0.09** | **2.13** | **0.039** |
|  |  | **End of Semester** | | | | | | | | | | | |
|  |  | RNT (PTQ) | | | | Worry (PWSQ) | | | | Rumination (RRS) | | | |
|  |  | *b* | *se* | *t* | *p* | *b* | *se* | *t* | *p* | *b* | *se* | *t* | *p* |
| **Main effects** |  |  |  |  |  |  |  |  |  |  |  |  |  |
| Trait Measure |  | 0.21 | 0.09 | 2.40 | 0.021 | 0.15 | 0.09 | 1.69 | 0.099 | 0.06 | 0.10 | 0.59 | 0.561 |
| RNT Type |  | **0.30** | **0.04** | **7.03** | **< 0.001** | **0.30** | **0.04** | **6.96** | **< 0.001** | **0.31** | **0.04** | **7.18** | **< 0.001** |
| Trait Measures x RNT Type | | 0.07 | 0.04 | 1.64 | 0.102 | **0.20** | **0.04** | **4.62** | **< 0.001** | **0.18** | **0.04** | **4.04** | **< 0.001** |
| **Simple slopes** |  |  |  |  |  |  |  |  |  |  |  |  |  |
| Rumination ~ Trait Measure | | **0.21** | **0.09** | **2.40** | **0.021** | 0.15 | 0.09 | 1.69 | 0.099 | 0.06 | 0.10 | 0.59 | 0.561 |
| Worry ~  Trait Measure | | **0.28** | **0.09** | **3.19** | **0.003** | **0.35** | **0.09** | **4.00** | **< 0.001** | **0.23** | **0.10** | **2.43** | **0.020** |

| Supplemental Table 3. Summary of univariate worry and rumination paradigm brain  models | | | | | |
| --- | --- | --- | --- | --- | --- |
| Univariate Model | Region | N voxels | peak x | peak y | peak z |
| RNT (Worry + Rumination - Neutral) | | |  |  |  |
|  | PCC/Precuneus | 161 | -0.3 | +66.7 | 34.2 |
|  | R. IPL | 18 | -38.1 | +46.1 | 41 |
|  | L. IPL | 15 | +44.4 | +42.6 | 41 |
|  | L. IPL | 13 | +30.7 | +49.5 | 44.4 |
| Worry - Neutral | |  | |  |  |
|  | PCC/Precuneus | 131 | -0.3 | +66.7 | 34.2 |
|  | L. IPL | 35 | +47.9 | +46.1 | 51.2 |
|  | R. MFG | 10 | -31.2 | -15.8 | 61.4 |
|  |  |  |  |  |  |
| Rumination - Neutral | |  | |  |  |
|  | PCC/Precuneus | 115 | +3.2 | +52.9 | 24 |
|  | R. IPL | 19 | -38.1 | +46.1 | 37.6 |
|  |  |  |  |  |  |
| Intensity Amplitude Modulation | |  |  |  |  |
|  | PCC/Precuneus | 52 | 3.2 | 52.9 | 30.8 |
| *Cluster size corrected at p = 0.001and a = 0.05. PCC = posterior cingulate cortex; R. = right; L = left; IPL = inferior parietal lobule; MFG = middle frontal gyrus* | | | | | |

| Supplemental Table 4. IAPS negative-neutral univariate contrast | | | | | |
| --- | --- | --- | --- | --- | --- |
| Region | N voxels | peak x | peak y | peak z |  |
| Bilateral mPFC | 50 | -0.3 | -60.5 | 24.0 |  |
| Left Lateral Occipital | 20 | +51.3 | +73.6 | +3.6 |  |
| Left Amygdala | 12 | +20.4 | +4.8 | -13.4 |  |

*Cluster corrected at p = 0.001, a = 0.05; mPFC= medial prefrontal cortex*

| Supplemental Table 5. RSA Searchlight: RNT cluster size and peak coordinates | | | | |
| --- | --- | --- | --- | --- |
|  | N voxels | peak x | peak y | peak z |
|  | 43475 | -44 | -7.0 | -21 |
|  | 37317 | +22.0 | 30 | +2.0 |
|  | 21359 | -16.0 | -35.0 | 60 |
|  | 12524 | +59.0 | 34 | +6.0 |
|  | 6490 | -64.0 | 34 | +6.0 |
|  | 3041 | -40 | -7.0 | 50 |
|  | 2124 | 59 | -3.0 | 43 |
|  | 1788 | -2.0 | -56.0 | -18 |
|  | 1779 | -47.0 | -11.0 | -35 |
|  | 1765 | +32.0 | -28 | -8.0 |
|  | 1735 | -47.0 | +58.0 | -28 |
|  | 1365 | -37.0 | -59 | +2.0 |
|  | 1105 | +42.0 | -18.0 | -18 |
|  | 1002 | -1.0 | +27.0 | 43 |
|  | 734 | -67.0 | 17 | -4.0 |
|  | 711 | -44.0 | 64 | -8.0 |
|  | 708 | -44.0 | 38 | -1.0 |
|  | 684 | -9.0 | +20.0 | 67 |
|  | 658 | -54.0 | +75.0 | 19 |
|  | 649 | -6.0 | -8.0 | 36 |
|  | 561 | -51.0 | 23 | -8.0 |
|  | 492 | 59 | -4.0 | 13 |
|  | 475 | -47.0 | +40.0 | -16 |
|  | 466 | +4.0 | +34.0 | 36 |
|  | 357 | +19.0 | -46.0 | 36 |
|  | 295 | -40.0 | +54.0 | -15 |
|  | 295 | +18.0 | +68.0 | 23 |
|  | 292 | +11.0 | 85 | -8.0 |
|  | 259 | -20.0 | +10.0 | 40 |
|  | 232 | -9.0 | -4.0 | 74 |
|  | 232 | -9.0 | +34.0 | 77 |
|  | 222 | -30.0 | -49 | -4.0 |
|  | 216 | +25.0 | 92 | -8.0 |
|  | 210 | -23.0 | 75 | -1.0 |
|  | 196 | -40.0 | +27.0 | 23 |
|  | 195 | -2.0 | -66 | -4.0 |
|  | 195 | +42.0 | +78.0 | 19 |
|  | 183 | -2.0 | -35.0 | -11 |
|  | 183 | +53.0 | -11 | +6.0 |
|  | 168 | -9.0 | +20.0 | 40 |
|  | 168 | +8.0 | +37.0 | 50 |
|  | 148 | -30.0 | 34 | -1.0 |
|  | 148 | -30.0 | 20 | +9.0 |
|  | 148 | +22.0 | -56.0 | 26 |
|  | 147 | -37.0 | +54.0 | -38 |
|  | 120 | -23.0 | 54 | -1.0 |
|  | 120 | -40.0 | +23.0 | 16 |
|  | 90 | -54.0 | 61 | -4.0 |
|  | 90 | -2.0 | 92 | -1.0 |
|  | 90 | +35.0 | -49.0 | 16 |
|  | 84 | -9.0 | +54.0 | -42 |
|  | 84 | -20.0 | +51.0 | -38 |
|  | 84 | -40.0 | +58.0 | -35 |
|  | 84 | +49.0 | -18.0 | -25 |
|  | 84 | -51.0 | -39.0 | -15 |
|  | 84 | -47.0 | 27 | -4.0 |
|  | 84 | -9.0 | 95 | -1.0 |
|  | 84 | -44.0 | 51 | +9.0 |
|  | 84 | +18.0 | 16 | +9.0 |
|  | 84 | -44.0 | -35.0 | 16 |
|  | 84 | +32.0 | -46.0 | 19 |
|  | 84 | -6.0 | +3.0 | 30 |
|  | 84 | -33.0 | -46.0 | 40 |
|  | 84 | -37.0 | -39.0 | 43 |
|  | 72 | -30.0 | +23.0 | 19 |
|  | 72 | +49.0 | -49.0 | 19 |
|  | 72 | -68.0 | +37.0 | 36 |
|  | 64 | -57.0 | +13.0 | -15 |
|  | 64 | -47.0 | 82 | -8.0 |
|  | 64 | -47.0 | -42 | +2.0 |
|  | 63 | -6.0 | 95 | -4.0 |
|  | 63 | -6.0 | -15 | -1.0 |
|  | 63 | -51.0 | -32.0 | 33 |

Supplemental Table 6. Statements from the fMRI task

| **Neutral Statements (*all subjects saw*)** | |
| --- | --- |
|  | I sometimes think about what it feels like to wash my hair |
|  | I sometimes think about office supplies |
|  | I sometimes think about what it feels like to throw a ball |
|  | I sometimes think about sorting laundry |
|  | I sometimes think about the layout of the grocery store |
|  | I sometimes think about the taste of water |
|  | I sometimes think about the different types of clouds |
|  | I sometimes think about how to care for a plant |
|  | I sometimes think about how a bicycle works |
|  | I sometimes think about which paint color suits my room |
|  | I sometimes think about the different creatures in the ocean |
|  | I sometimes think about different routes to take to class |
|  | I sometimes think about flipping through magazines |
|  | I sometimes think about how someone develops common sense |
|  | I sometimes think about different styles of sock |
| **Example Worry Statements (*subject-specific*)** | |
|  | I am worried that I won't be able to find a job for the summer. |
|  | I am worried she'll get tired of me |
|  | I am worried my parents will give me less support |
|  | I am worried that I am wasting life |
|  | I am worried that my mom will be lonely when my brother leaves for college. |
|  | I am worried I will not get the grades I need |
|  | I am worried that I am a bad writer and cannot do well in future english essays. |
|  | I am worried that I might hurt myself playing rugby. |
|  | I am worried that I take an initiative and I'll fail |
|  | I am worried that I will be average |
|  | I am worried that my mom and I will have to move back to my abuela's house next year |
| **Example Rumination Statements (*subject-specific*)** | |
|  | I often think back on how anxiety provoking meeting them for the first time was. |
|  | I often think back on how bad it was that I wasn't prepared for class when I should have been |
|  | I often think back on letting the good ones go. |
|  | I often think back on how I could have put more effort into past relationships. |
|  | I often think back on how perfectionistic on assignments I am |
|  | I often think back on anything embarrassing I say in front of the class. |
|  | I often think back on how worthless things made me feel. |
|  | I often think back on how poorly my dad treated our family. |
|  | I often think back on what I could have done to do better on an exam |
|  | I often think back on being too involved in activities to make time for myself |

| **Supplemental Table 7. Large Language Model Statement Classification Examples** | | | |
| --- | --- | --- | --- |
|  | **Participant's statement provided to the model  (worry/rum stem removed)** | **Model classification** | **Model rationale for statement classification** |
| **correctly classified ruminations** | …how my sister and I used to fight a lot. | Rumination | First, the statement focuses on a past event, which is the frequent fighting between the speaker and their sister. Reflecting on past conflicts can lead to negative thoughts and emotions, as the speaker may ruminate on the reasons for the fights and regret past actions. Therefore, this statement meets the definition for rumination. |
|  | …when I looked stupid infront of the entire class | Rumination | First, the statement focuses on an event in the past, which is looking stupid in front of the entire class. Reflecting on this negative experience could lead to negative thoughts and emotions. Therefore, this statement is focused on a past occurrence that could lead to negative thoughts and emotions, meeting the definition for rumination. |
|  | …how much money I wasted on useless things. | Rumination | First, the speaker in the statement is reflecting on the past, specifically on the amount of money they have spent. This is a past-focused statement. Additionally, the use of the word "useless" implies negative emotions and thoughts towards the past spending. Therefore, this statement meets the definition for rumination. |
| **correctly classified worries** | …that my friends do not truly care about me. | Worry | First, the speaker in the statement expresses a concern about their friends' feelings towards them. This is a future-oriented statement as it implies a potential negative outcome that has not yet occurred. Additionally, the statement involves negative thoughts and emotions related to the speaker's relationships with their friends.  Therefore, this statement meets the definition for worry as it is focused on a future event that could bring negative emotions and is concerned with the speaker's relationships. |
|  | …that I will never get rid of my headaches | Worry | First, the speaker in the statement notes a hypothetical situation where they will never get rid of their headaches. Since the absence of headaches in the future is an uncertain event, it is a future statement. Additionally, the potential of having headaches indefinitely could evoke negative thoughts and emotions for the speaker of the statement. Therefore, this statement is future-focused on an undesirable outcome, meeting the definition of worry. |
|  | …that I will not get a high enough MCAT score to get into med school. | Worry | First, the speaker in the statement notes a hypothetical situation where they will not get a high enough score on the MCAT exam. Since the MCAT exam has yet to be taken, it is a future statement. Additionally, not getting a high enough score could be a negative outcome, as it may prevent the speaker from getting into med school. Because this statement is focused on a future event that could bring negative emotions, it meets the definition for worry. |
| **incorrect worry classifications** | …that I push people away too much. | Rumination | First, the statement is about a potential behavior of the speaker, which is pushing people away. This behavior has already occurred in the past, as it is a characteristic trait. Reflecting on this behavior and the potential negative impact it may have on relationships could lead to negative thoughts and emotions. Therefore, this statement is focused on a past behavior that could lead to negative thoughts and emotions, meeting the definition for rumination. |
|  | …that I am overweight | Rumination | First, the statement is about a personal perception of oneself, which is a present-day concern. However, it is not focused on a specific future event or outcome. Instead, it is a continuous thought about one's current state. While this thought can lead to negative emotions and potentially cause worry about future health consequences, it is more accurately described as a self-perception or rumination. |
|  | …that all my hard work was for nothing. | Rumination | First, the statement is about a past event, as the speaker is reflecting on their hard work. Additionally, the statement implies a negative outcome, as the speaker believes that their efforts may have been in vain. This statement is focused on a past occurrence that could lead to negative thoughts and emotions, meeting the definition for rumination. |
| **incorrect rumination classification** | …how I can be less awkward in an interaction | Worry | The statement is about a personal concern for improving a trait (being less awkward) in interactions, which is a future-oriented goal. This statement is not about a specific past event or a negative outcome, but rather a desire to change a characteristic. Therefore, this statement is an example of worry, as it involves negative thoughts about the future. |
|  | …messing up a kiss | Worry | The statement refers to a hypothetical situation where the speaker might mess up a kiss. Since this event has not yet occurred, it is a future statement. Additionally, the potential for messing up a kiss could evoke negative thoughts and emotions for the speaker, such as embarrassment or regret. Therefore, this statement is future-focused on an undesirable outcome, meeting the definition of worry. |
|  | …how my financial situation would be better if I went to a different college | Worry | First, the speaker in the statement is considering a hypothetical situation where they went to a different college. This is a future statement as the action of going to a different college has not yet occurred. Additionally, the statement implies a negative perception of the current financial situation and a desire for improvement. Since this statement is focused on a future event that could bring about negative thoughts and emotions related to the current situation, it meets the definition for worry. |
